# Mitochondrial-encoded peptide MOTS-c prevents pancreatic islet cell senescence to delay diabetes

**DOI:** 10.1038/s12276-025-01521-1

**Published:** 2025-08-25

**Authors:** Byung Soo Kong, Hyunsuk Lee, Sehi L’Yi, Serin Hong, Young Min Cho

**Affiliations:** 1 Division of Endocrinology and Metabolism, Department of Internal Medicine, Seoul National University Hospital, Seoul, Korea; 2 Department of Molecular Metabolism, T.H. Chan School of Public Health, Harvard University, Boston, MA 02115 USA; 3 Broad Institute of MIT and Harvard, Cambridge, MA 02142 USA; 4 Department of Internal Medicine, Seoul National University College of Medicine, Seoul, Korea; 5 Genomic Medicine Institute, Medical Research Center, Seoul National University College of Medicine, Seoul, Korea; 6 Department of Biomedical Informatics, Harvard Medical School, Harvard University, Boston, MA 02115 USA

**Keywords:** Type 2 diabetes, Type 1 diabetes, Diagnostic markers

## Abstract

Mitochondria are crucial for cell survival and function, partly through peptides encoded by the mitochondrial genome. Although mitochondrial dysfunction is a hallmark of age-related diseases and senescence, the role of mitochondrial-genome-encoded peptides in pancreatic β-cell senescence during type 1 and type 2 diabetes pathogenesis is largely unexplored. Here we show that MOTS-c levels decrease with aging and senescence in pancreatic islet cells. Treating aged C57BL/6 mouse pancreatic islets with MOTS-c reduced pancreatic islet senescence by modulating nuclear gene expression and metabolites involved in β-cell senescence. MOTS-c treatment improved pancreatic islet senescence and glucose intolerance in S961-treated C57BL/6 and in nonobese diabetic mice. In humans, circulating MOTS-c levels are lower in type 2 diabetes patients compared with healthy controls. Our findings suggest that mitochondrial-encoded MOTS-c regulate pancreatic islet cell senescence and that MOTS-c could act as a senotherapeutic agent to prevent pancreatic islet cell senescence and diabetes progression.

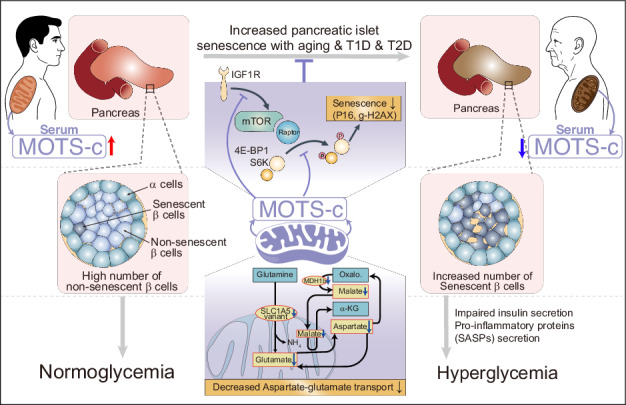

## Mitochondrial peptide MOTS-c reduces pancreatic cell senescence

Diabetes is a condition where the body struggles to manage blood sugar levels due to issues with insulin, a hormone produced by the pancreas. Researchers have found that aging and stress can cause pancreatic cells to become ‘senescent’, meaning they stop working properly. This study explores how a small protein called MOTS-c, made by mitochondria, might help these cells function better. Researchers found that MOTS-c levels decline with aging in human serum and in publicly available datasets from both humans and mice. Researchers used mice to test if MOTS-c could reduce cell aging in the pancreas. They treated different groups of mice with MOTS-c and observed its effects on pancreatic cells. They also studied mouse cells to see if similar benefits occurred. The study found that MOTS-c treatment reduced signs of aging in pancreatic cells and improved their function. Results suggest that MOTS-c could be a promising treatment for diabetes by helping pancreatic cells stay healthy longer. This summary was initially drafted using artificial intelligence, then revised and fact-checked by the author.

## Introduction

Diabetes is a metabolic disorder marked by a loss of pancreatic β-cell mass and function^1,2^. While the two major types of diabetes, type 1 and type 2 (hereafter T1D and T2D, respectively), have their differences in pathogenesis, they share senescence of pancreatic β-cells as a common pathogenetic mechanism^1,2^. Senescence in pancreatic β-cells exhibits altered mitochondrial activity, mitochondrial protein expression levels and decreased mitochondrial DNA (mtDNA) copy number^3,4^. In fact, dysfunction in mitochondria and mitochondrial factors, also known as the mitochondrial dysfunction-associated senescence (MiDAS)^5^, can induce senescence and maybe a potential factor contributing to senescence in T1D and T2D pancreatic β-cells.

Mitochondria possess a circular genome that was traditionally thought to host only 13 protein-coding genes. Recent research has uncovered short open-reading frames that encode bioactive mitochondrial-derived peptides within the mitochondrial genome^6–9^. One such peptide, MOTS-c (mitochondrial ORF of the 12S rRNA type-c), plays a role in regulating metabolic homeostasis, partly by regulating AMPK and mTOR pathways^7,8,10^. The expression of MOTS-c is age-dependent and has been detected in multiple tissues, including skeletal muscle, T cells and in systemic circulation^6–10^ and the pancreas^7,11^. In fact, systemic administration of MOTS-c has been shown to reverse diet-induced insulin resistance by enhancing glucose uptake in the skeletal muscle of C57BL/6 mice and to mitigate T cell-mediated insulin deficiency by ameliorating autoimmune pancreatitis in nonobese diabetic (NOD) mice^7,8^. However, the specific functions and impacts of insulin-sensitizing mitochondrial-encoded MOTS-c in pancreatic islet cells related to T1D or T2D, as well as its role in cellular senescence, are still not fully understood.

Chronological aging and metabolic insults induce the accumulation of senescent β-cells, increasing the risk of insulin resistance^1,2,12,13^. β-cell senescence has been reported to be induced by chronological aging^12^ and by systemic treatment with the insulin-receptor antagonist, S961, which acutely induces of β-cell-specific senescence and hyperglycemia in mice^1,12^. Removal of S961 reverses the β-cell senescence in S961-treated mice^12^. Furthermore, senescent β-cells accumulate in NOD mice, a T1D mouse model, and the removal of senescent β-cells protects against T1D^2,14^. These reports suggest that three different senescent β-cell animal models may share pathogenic similarities involving transcriptome or metabolome, which could be targeted simultaneously. MOTS-c has demonstrated therapeutic effects by enhancing physical performance in aged mice^9^, reducing insulin-resistance in high-fat diet-fed mice^8^ and preventing pancreatic infiltration by T cells in NOD mice^7^. However, the role of MOTS-c in β-cell senescence has yet to be discovered.

In this study, we hypothesize that MOTS-c could reduce senescence in pancreatic islets and β-cells. Our analysis showed that MOTS-c treatment substantially reduced pancreatic islet and β-cell senescence in three different mouse models, which are chronological aged (~60–90-week-old) C57BL/6 mice, NOD mice and S961-treated β-cell-specific senescence-induced hyperglycemic C57BL/6 mouse model. Furthermore, MOTS-c regulates metabolite transport process (aspartate–glutamate) to reduce glutaminolysis-dependent senescence in pancreatic islet and Min6 cells to delay diabetes.

## Methods

### Cell lines

Min6 and NIT-1 cells were grown in Dulbecco’s modified Eagle medium (DMEM) supplemented with 20% fetal bovine serum (FBS). HEK293FT was grown in DMEM supplemented with 10% FBS. All cells except Min6 (ref. ^15^) were purchased from ATCC. The cells were incubated in a humidity and atmospheric oxygen-controlled environment at 37 °C, 5% CO_2_ incubator, were utilized up to ten passages. For glutamine treatment, FBS-supplemented culture media was removed and replaced with glutamine-pyruvate and sodium-pyruvate depleted XF DMEM media pH 7.4 (Agilent). Glutamine (Agilent) was added to the cells depending on the condition.

### Animals

The mice were kept in a 12-h light–dark cycle, were fed a normal chow diet for various durations and were kept in a room with a temperature between ~22.3–22.8 °C. NOD or C57BL/6 mice were conducted in the specific pathogen-free environment in the animal facility at the Seoul National University Hospital with approval of Animal Care and Use Committee (IACUC nos. 17-0095-C1A1 and 18-0144-S1A1). The 6-week-old NOD (female, *n* = 18 per group) were purchased from the Jackson laboratory, and upon arrival, NOD mice had 1 week of stabilization before any experimental procedure. The 12-, 28-, 60- and 90-week-old C57BL/6 mice were acquired from Joongah Bio. Then, mice were weight- and random blood glucose-matched for further analyses.

### Human studies

A total of 45 patients with T2D fulfilling diagnostic criteria for T2D and 19 healthy controls were enrolled from the Division of Endocrinology and Metabolism, Department of Internal Medicine, Seoul National University Hospital (IRB nos. 1808-151-967, H-1112-030-388 and 1911-059-1078). Definition of T2D were based on the American Diabetes Association diagnostic criteria using fasting glucose, 2 h glucose and HbA1c^16^. The average age and percentage of female participants were 29.1 ± 6.8 years and 26% in the healthy control group and 64.7 ± 7.9 years and 51% in the T2D group.

### Ethics approval

This study was approved by the Ethics Committee of Seoul National University Hospital, Korea, and all methods were performed in accordance with the relevant guidelines and regulations. Written informed consent was obtained from all participants.

### Real-time PCR

Total RNA was purified using the RNeasy Mini Kit (Qiagen) as per manufacturer’s protocol. Complementary DNA was synthesized by reverse transcription of 1 μg of total RNA using High Capacity cDNA Reverse Transcription Kit (Applied Biosystems, cat. no. 4368814) following manufacturer’s instructions. Quantitative real-time PCR was performed in 20 μl of reaction mixture containing 1 μl of cDNA, 10 pM of each primer (*Slc1a5*, *Slc1a5* variant *Gls1*, *Gls2*, *Cdkn2a*, *Cdkn1a*, *Cd38*, *Igf1r*, *Il-1b*, *Il-6* and *Il-10*) and 10 μl of SYBR Select Master Mix (Thermo Fisher Scientific) using Applied Biosystems 7500 Real-Time PCR System (Thermo Fisher Scientific). The primers for *Slc1a5*, *Slc1a5* variant, *Gls1*, *Gls2*, *Cdkn1a*, *Cdkn2a*, *Cd38*, *Igf1r, Il-1b, Il-6* and *Il-10* were previously reported^17–22^. The primers for *ephrin-A5* (forward: AACAGCAGCAACCCCAGATTC, reverse: AAGCATCGCCAGGAGGAACAGTAG) and *EphA5* (forward: GCAAGTATTATGGGGCAGTTCG, reverse: ATAGAGAGCAGCAGGGCAATCC). Mouse *Mdh1* (forward: CGTAAAGTCCCTTCTTGACTTGC, reverse: CGCTTCATAAGCATGCCACC), mouse Mdh1b (forward: CTTCAAGGATGCACTGTGGCT, reverse: TGGACCCTTGAGTGGATGCTA), mouse Mdh2 (forward: CGGAAGTGCAGCCCAGAATA, reverse: CTGGATACGCACCCCCAAG), mouse *Got1* and mouse *Got2* (forward: CCGTTATCATCCCGGTAGGC, reverse: TGGATAGCTGGTGCCTGTTC) were used. For noncell-autonomous assay, the pancreatic islet cells isolated from 28-week-old C57BL/6 mice were treated with MOTS-c (10 μM, 6 h) in the presence or absence of H_2_O_2_ (200 μM, 6 h). Then, media were replaced with fresh cell culture media for 18 h. After 24 h, the conditioned media were collected from each condition to treat new pancreatic islet cells. Finally, the cells were obtained to perform quantitative PCR (qPCR).

### Western blotting

Whole lysates, mitochondrial or nuclear fraction were extracted with either lysis buffer containing a protease/phosphatase inhibitor cocktail or other appropriate kits as previously reported^6,7^. All western blot data are representative of at least three independent experiments.

### Virus production

HEK293FT cells were transfected with the VSVG envelope plasmid, retroviral packaging plasmids Gag-Pol and target plasmids. The OPTI-MEM medium was changed to FBS-supplemented media 8 h after transfection. The virus-containing supernatant was collected 48 h after transfection and spun and filtered to eliminate debris and cells.

### Transfection

Min6 and NIT-1 cells were transfected with either pLJM1-MOTS-c, pLJM1-empty, pGenLenti-*Cdkn2a* (Genscript) or pGenLenti-empty (SH016)-vectors in 2.0 × 10^6^ cells in six-well-plates using Neon transfection system (Thermo Fisher Scientific) as previous reported^6–8^ or in RPMI containing polybrene (10 μg ml^−1^; TR-1003-G, Millipore). For viral transfection with polybrene, the cells were transduced with lentivirus by centrifugation at 1,500 rpm for 45 min at 37 °C. After an 18-h incubation, the medium was changed to fresh culture medium containing selection reagent (pLJM1, pGenLenti: puromycin) and selected for 72 h.

### In vivo animal studies

MOTS-c peptides were synthesized as previously^7,8^. Exendin-4 was purchased from Abcam. The NOD mice were treated with either vehicle (nonspecific scrambled MOTS-c peptide), MOTS-c (0.5 mg kg^−1^ per day, intraperitoneally (i.p.))^7^, exendin-4 (2 μg kg^−1^, two times a day, i.p.) or both^23^. Diabetic phenotype of NOD and S961-treated C57BL/6 mice were assessed based on the previous study^1,7^. Intraperitoneal glucose tolerance tests (IPGTTs) were performed at 14-week-old for NOD mice and at 12 days post mini-pump implantation for S961-treated mice. Before the IPGTT, NOD or C57BL/6 mice were transferred to clean cages with no food for fasting overnight (12 h). The mice were weighed to calculate the volume of 20% glucose solution required for intraperitoneal injection (10 µl × body weight, in grams), and random blood glucose levels were measured for 0 min. Before and after 20% glucose injection, blood was collected from the submandibular vein^7^. The collected serum was stored at −80 °C to analyze insulin content within 2 weeks (Alpco). After sacrifice, pancreas tissues were collected from mice to be stored in OCT compound or fixed in paraformaldehyde for further analyses.

### Islet β-cell isolation

The mice were killed, and freshly prepared collagenase P (Roche) solution (0.5 mg ml^−1^) was injected into the pancreas via the common bile duct. The perfused pancreas was digested at 37 °C for 13 min with a short vortex every 3 min, and the islets were processed with gradient centrifugation with Pancoll human solution (PAN-Biotech) and then handpicked under a stereoscopic microscope. Then, the cells were dispersed into a single cell suspension in DMEM media supplemented with 20% FBS.

### S961 treatment in vivo

Vehicle (PBS) or 20 nmol S961 (Phoenix Pharmaceuticals) with scrambled MOTS-c or MOTS-c (0.5 mg kg^−1^ per day) was loaded into Alzet osmotic pump 2001 or 2004 and implanted subcutaneously on the back of the 28-week-old C57BL/6 mice^1,24,25^ and changed weekly for a total of 2 weeks for 2001. For Alzet osmotic pump 2004, no additional replacement surgery was performed.

### In-house circulating MOTS-c ELISA test

The enzyme-linked immunosorbent assay (ELISA) method was used and modified from the previous study to test MOTS-c levels in human serum^7^. Information of human patients, sampling dates, sex, age and MOTS-c levels are listed in Supplementary Fig. 1 and Supplementary Table 2.

### Immunofluorescence analyses on pancreas tissue samples

C57BL/6 and NOD mice were sacrificed, and pancreas were obtained and preserved either in 4% paraformaldehyde or in OCT compound for frozen cryotome sectioning. Hematoxylin and eosin (H&E) staining was performed in pancreas tissues preserved in 4% PFA. Pancreas embedded in frozen block were prepared in 5 μM cross sections for immunofluorescence staining. The sections were blocked with 5% goat serum (Jackson ImmunoResearch, 005-000-121) in antibody diluent (Dako) for 30 min and incubated overnight at 4 °C with following antibodies: insulin, glucagon, γ-H2AX, MOTS-c or IL-1β (1:100 of antibody concentration). MOTS-c antibody was preincubated with poly-l-lysine 4 °C overnight (Sigma) to avoid nonspecific immunocytochemical reactions^26^. After washing with TBST, slides were incubated for 2 h with appropriate fluorochrome-attached secondary antibody (Jackson ImmunoResearch). The slides were rinsed and analyzed after mounting coverglass. Then, the images were acquired by using confocal microscopes (TCS-SP8, Leica, magnification 20×; Celloger Mini Plus, Curiosis, magnification 10×). For quantification, the islet images were captured systematically covering the whole section in confocal mode on a microscope to evaluate every cluster of insulin-stained cells (three to seven cells) or islets (eight or more cells) per section was evaluated for γ-H2AX, MOTS-c or IL-1β intensity (A.U.); the sections were coded and read blindly.

### SA-β-gal staining

SA-β-gal staining procedure was followed as described previously^27,28^. Briefly, OCT-embedded, snap frozen, unfixed tissues were cryosectioned at thickness of 10 μm and stored at −80 °C until use. For SA-β-gal staining of frozen tissue sections (*n* = 10 per group), pancreas sections were thawed, fixed and stained using a senescence β-galactosidase staining kit (Cell Signaling, cat. no. 9860). For live cell SA-β-gal-staining using FACS, live pancreatic islet cells isolated from C57BL/6 mice were stained with CellEvent Senescence Green Flow Cytometry Assay Kit (Thermo, cat. no. C10840) by following manufacturer’s protocol.

### Flow cytometry and intracellular staining of pancreatic β-cells

Isolated mouse islets were dispersed using EDTA-PBS for 15 min at 37 °C and were resuspended in FACS buffer (0.5% BSA (Millipore) in PBS). Using FACS Aria or Canto (BD), the cells were gated according to forward scatter and the percentage of β-cells by insulin staining ranged from ~85–90%, as previously reported^29^. For evaluation of β-gal activity, a fluorescent substrate for flow cytometry assay kit was used (Thermo, cat. no. C10840) following the manufacturer’s protocol. For other antibodies, the cells were stained with adequate antibodies, washed and analyzed in FACS buffer. For intracellular cytokine staining, the cells were stained with surface markers (Alexa647 anti-mouse Igf1r, BD, cat. no. 558588) and then fixed and permeabilized using Intracellular Fix/Perm or transcription factor Fix/Perm buffer set (eBioscience). After permeabilization, intracellular staining antibodies (PE-anti-mouse IL-1β, eBioscience, cat. no. 12-7114-82; APC-CXCL10, LSBio, cat. no. LS-C738568-100; V450-anti-mouse IL-6, BD, cat. no. 561376; Alexa488-Insulin, Cell Signaling, cat. no. 9016; Alexa488-P21 or Alexa647-P21, Abcam, cat nos. ab237264 and ab237265) with different fluorochromes were added for intracellular staining. The cells were analyzed by flow cytometry, and the data were analyzed with Flowjo software (Treestar, Ashland).

### Microarray

RNA purity and integrity were evaluated by ND-1000 Spectrophotometer (NanoDrop), Agilent 2100 Bioanalyzer (Agilent Technologies). RNA labeling and hybridization were performed by using the Agilent One-Color Microarray-Based Gene Expression Analysis protocol (Agilent Technology, V 6.5, 2010). Briefly, 100 ng of total RNA from each sample was linearly amplified and labeled with Cy3-dCTP. The labeled cRNAs were purified by RNAeasy Mini Kit (Qiagen). The concentration and specific activity of the labeled cRNAs (picomole Cy3 per microgram cRNA) were measured by NanoDrop ND-1000(NanoDrop). A total of 600 ng of each labeled cRNA was fragmented by adding 5 μl 10× blocking agent and 1 μl of 25× fragmentation buffer and then heated at 60 °C for 30 min. Finally, 25 μl 2× GE hybridization buffer was added to dilute the labeled cRNA. A total of 40 μl of hybridization solution was dispensed into the gasket slide and assembled to the Agilent SurePrint G3 Mouse GE 8X60K, V2 Microarrays (Agilent). The slides were incubated for 17 h at 65 °C in an Agilent hybridization oven, then washed at room temperature by using the Agilent One-Color Microarray-Based Gene Expression Analysis protocol (Agilent Technology, V 6.5, 2010). The hybridized array was immediately scanned with an Agilent Microarray Scanner D (Agilent Technologies). The microarray results were extracted using Agilent Feature Extraction software v11.0 (Agilent Technologies). The array probes that have Flag A in samples were filtered out. The selected gProcessedSignal value was transformed by logarithm and normalized by quantile method. The statistical significance of the expression data was determined using independent *t*-test and fold change in which the null hypothesis was that no difference exists among groups. The false discovery rate (FDR) was controlled by adjusting the *P* value using the Benjamini–Hochberg algorithm. For a differentially expressed gene (DEG) set, a hierarchical cluster analysis was performed using complete linkage and Euclidean distance as a measure of similarity. The microarray data were processed with Gene Ontology (GO) and Kyoto Encyclopedia of Genes and Genomes (KEGG) and are included in Supplementary Table 1.

### Metabolic flux analyses

Murine pancreatic islet cells were obtained from NOD mice after the treatment of either vehicle or MOTS-c for 18 weeks. The isolated pancreatic islet cells from each group were plated onto Seahorse cell plates coated with Cell-Tak (Corning) to enhance cell attachment. The cells were analyzed with the Seahorse XFe24/96 Extracellular Flux analyzer (Agilent) to determine real-time oxygen consumption rate and extracellular acidification rate and normalized the Seahorse measurements by counting cell number with Biotek citation 1/5, as previous reported^7^.

### Targeted metabolomics using LC–MS/MS

The AbsoluteIDQ p180 kit (Biocrates) with a liquid chromotography–tandem mass spectrometry (LC–MS/MS) system (Sciex 6500 + QTRAP) was used to quantify metabolites in aged pancreatic islet cells. The LC–MS/MS system was also used to quantify 188 metabolites in aged pancreatic islet cells, allowing the concurrent high-throughput detection and quantification of metabolites in samples. The sample preparation and LC–MS/MS analytical procedure was performed by Seoul National University Hospital Metabolomics Core Facility and are described in detail in previous reports^30^. The average percent coefficient of variance (CV) of analytes ranged between 15–30% CV range and the concentrations of limit of detection are listed in Supplementary Table 3. The quantified measurements were analyzed by MetaboAnalyst program^31^.

### TEM

The cells were fixed overnight in a mixture of cold 2.5% glutaraldehyde in 0.1 M phosphate buffer (pH 7.2) and 2% paraformaldehyde in 0.1 M phosphate or cacodylate buffer (pH 7.2). These cells were post-fixed for 1.5 h in 2% osmium tetroxide in 0.1 M phosphate or cacodylate buffer for 1.5 h at room temperature. The samples were then washed briefly with deuterated H_2_O_2_, dehydrated throughout a graded 50, 60, 70, 80, 90, 95 and 100% ethanol (X2) series, infiltrated by using propylene oxide and EPON epoxy resin mixed (Embed 812, Nadic methyl anhydride, poly Bed 812, dodecenylsuccinic anhydride, dimethylaminomethyl phenol, Electron Microscopy Polysciences, and finally embedded with only epoxy resin. The epoxy resin-mixed samples were loaded into capsules and polymerized at 38 °C for 12 h and 60 °C for 48 h. The sections for light microscopy were cut at 500 nm and stained with 1% toluidine blue for 45 s on a hot plate at 80 °C. The thin sections were made using an ultramicrotome (RMC MT-XL) and collected on copper grid. Appropriate areas for thin sectioning were cut at 65 nm and stained with saturated 6% uranyl acetate and 4% lead citrate before examination with a transmission electron microscope (JEM-1400; Japan) at 80 Kv. The sections were coded and read blindly for mitochondrial size and measured by ImageJ software.

### Analysis of publicly available single-cell RNA-seq data

Raw reads from publicly available datasets were obtained as follows: GSE164471^32^, GSE163847^33^, GSE205853^34^, PXD014244^35^, GSE81608^36^, EGAD00001008777^37^, GSE130727^38^, GSE64553^39^, GSE72815^40^, GSE98440^41^, and GSE102004^42^.

### Quantification and statistical analysis

The data are presented as the mean ± standard error of mean (s.e.m.). Graphpad Prism was used for two-tailed *t*-test, one-way, two-way or repeated measures two-way analysis of variance (ANOVA) to assess significance between groups and Tukey’s multiple comparison was performed.

## Results

### MOTS-c declines with chronological aging in pancreatic β-cells

**Fig. 1:**
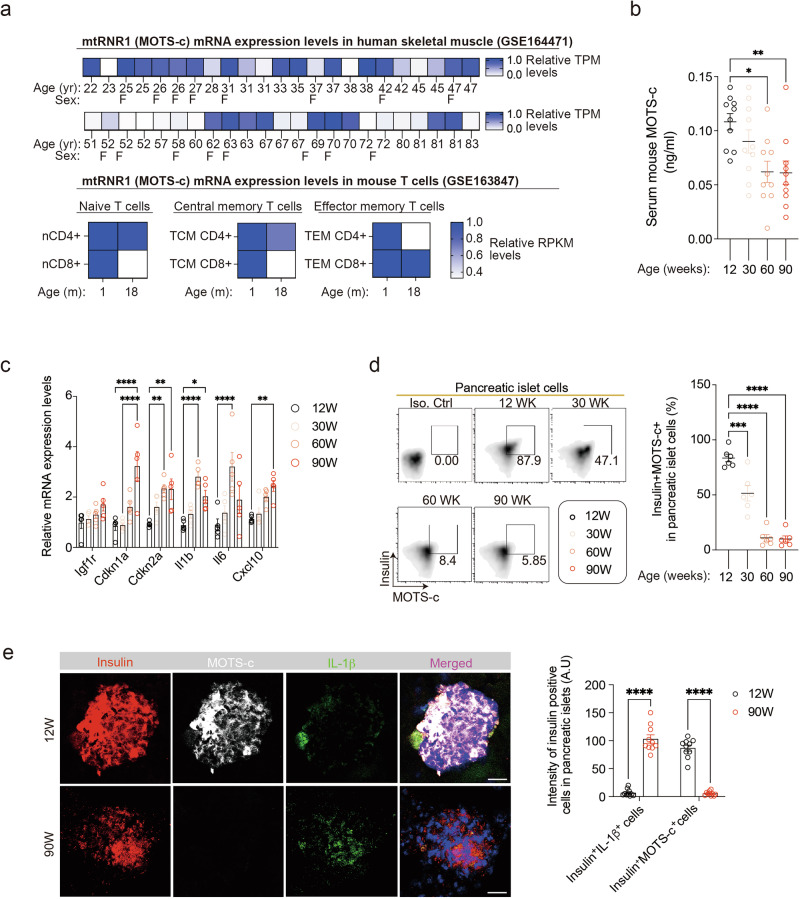
MOTS-c declines with chronological aging in β-cells. **a** mtRNR1 mRNA expression levels were analyzed using publicly available datasets (GSE164471, GSE163847) (Supplementary Table 4). Skeletal muscle transcriptome data (relative Transcripts per million, TPM, levels) from healthy individuals of varying ages (22–83 years) and sex (blank, male; F, female) were obtained from GSE164471. Mouse CD4^+^ and CD8^+^ T cell (naive, central memory and effector memory T cells) transcriptome data (relative TPM levels) from mice aged either 1 or 18 months were obtained from GSE163847 (Supplementary Table 4). In addition, mouse serum and pancreatic islet cells were isolated from C57BL/6 mice aged 12, 30, 60 and 90 weeks for various analyses, including MOTS-c ELISA, qPCR, immunofluorescence (IF) staining and flow-cytometry. **b** Using MOTS-c ELISA, serum MOTS-c levels were measured. MOTS-c levels decreased with age in 60- and 90-week-old C57BL/6 mice compared with 12-week-old mice (*n* = 10 per group; one-way ANOVA; error bars are the s.e.m.). *P < 0.01, **P < 0.05. **c** qPCR analysis was performed to compare mRNA levels of *Igf1r*, *Cdkn1a*, *Cdkn2a*, *Il-1b*, *Il-6* and *Cxcl10* in pancreatic islet cells isolated from mice aged 12, 30, 60 and 90 weeks (two-way ANOVA; the error bars are the s.e.m.). *P < 0.05, **P < 0.01, ****P < 0.0001. **d** Flow-cytometry was employed to compare intracellular MOTS-c and insulin in pancreatic β-cells isolated from 12- and 90-week-old mice (one-way ANOVA; the error bars are the s.e.m.). ****P < 0.0001. **e** IF staining of MOTS-c in insulin-positive pancreatic β-cells showed higher expression of MOTS-c in 12-week-old β-cells compared with 90-week-old β-cells. IL-1β-expressing β-cells were more prevalent in 90-week-old mice than in 12-week-old mice. The pancreatic sections were prepared as described; the representative data are from ten pancreas sections for each group (*n* = 10 per group). Scale bars, 50 μm; two-way ANOVA; the error bars are the s.e.m. ****P < 0.0001.

The coding sequence for the mitochondrial open reading frame of the 12S rRNA-c (MOTS-c) is located within the 12S rRNA gene (mtRNR1) of mtDNA^6–8,10^ and is closely linked to aging^9^. Given that MOTS-c function has been examined in skeletal muscle and T cells by us and our colleagues^7,8^, we investigated the expression levels of MOTS-c (mtRNR1) in publicly available datasets from human skeletal muscle^32^ and mouse T cell^33^. The analyses showed that mtRNR1 (MOTS-c) expression levels decrease in aged human skeletal muscle^32^ and in aged (18-month-old) mouse T cells^33^ compared with the young (Fig. 1a). To understand the temporal relationship between MOTS-c levels and chronological age, we collected mouse serum and pancreatic islets from 12-, 30-, 60- and 90-week-old C57BL/6 mice. The circulating mouse MOTS-c levels decreased with age in C57BL/6 mice, particularly in the 60- and 90-week-old mice compared with the 12-week-old mice (Fig. 1b). Moreover, as the mice aged, there was a gradual increase of senescent markers with age in the pancreatic islets, including elevated mRNA levels of *Igf1r*, *Cdkn1a*, *Cdkn2a*, *Il-1b*, *Il-6* and *Cxcl10* (Fig. 1c). Further analysis with flow-cytometry revealed that a decline in MOTS-c expression levels within insulin-positive β-cell population of the pancreatic islets as aging progressed from 12 to 90 weeks (Fig. 1d). To confirm the expression levels of MOTS-c in β-cells, we performed immunofluorescence staining for insulin, MOTS-c and IL-1β, a key cytokine associated with the senescence-associated secretory phenotype (SASP)^43^. In 12-week-old C57BL/6 mice, MOTS-c expression was higher, and there were fewer IL-1β-expressing cells in insulin-positive β-cells, whereas in the 90-week-old mice, pancreatic islets showed a complete loss of MOTS-c expression and a higher number of IL-1β-expressing cells in insulin-positive β-cells (Fig. 1e). Moreover, MOTS-c expression did not overlap with IL-1β-expression, suggesting that MOTS-c levels are negatively correlated with cellular senescence (Fig. 1e). These findings indicate an inverse relationship between MOTS-c and senescence/aging in β-cells.

### Systemic MOTS-c treatment prevents pancreatic β-cell senescence and pancreatic insulitis in NOD mice

**Fig. 2:**
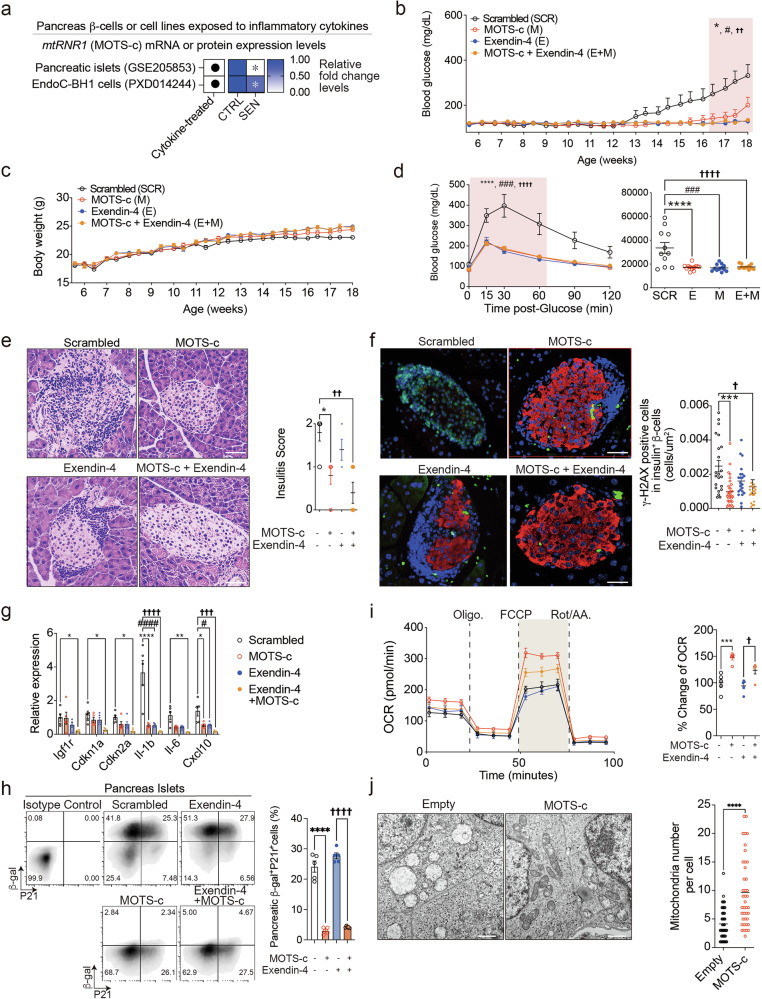
Systemic MOTS-c treatment prevents β-cell senescence and pancreatic insulitis in NOD mice. **a** The publicly available datasets were analyzed to assess mtRNR1 mRNA (GSE205853; fold change) and protein (PXD014244; fold change) levels in pancreatic β-cells or β-cell lines (EndoC-BH1 cells) with statistical significance indicated (*P < 0.05) (Supplementary Table 4). The 5-week-old NOD mice (*n* = 20 per group) were acquired and maintained for 2 weeks. The 7-week-old NOD mice were treated either with scrambled peptide (SCR), MOTS-c (M, 0.5 mg kg^−1^ per day, i.p.), exendin-4 (E, 2.0 µg kg^−1^, two times a day, i.p.) or both peptides (E + M) until 18 weeks of age (Supplementary Fig. 2a). **b**,**c** NOD mice were monitored for blood glucose levels (**b**) and body weight (**c**). **d** An IPGTT was performed at 14-weeks of age, and area under the curve (AUC) was analyzed. **e** After sacrifice, H&E staining was conducted in NOD pancreas sections to evaluate pancreatic insulitis in NOD mice treated with either scrambled, MOTS-c, exendin-4 or both. The pancreatic sections were prepared as described and representative data are from five pancreas sections per group are shown. Scale bars, 50 μm; repeated measures two-way ANOVA or two-way ANOVA; error bars are the s.e.m. **f** At 18 weeks, NOD pancreatic islet cells were isolated for immunofluorescence staining of insulin and γ-H2AX. The pancreatic sections were prepared as described and representative data are from ten pancreas with two sections for each group. Scale bars, 50 μm; two-way ANOVA; the error bars are the s.e.m. **g** The pancreatic islets from each group were assessed by qPCR analysis on *Igf1r*, *Cdkn1a*, *Cdkn2a*, *Il-1b*, *Il-6* and *Cxcl10*. Two-way ANOVA; error bars are the s.e.m. **h** Flow cytometry was conducted on β-gal and P21 in 18-week-old NOD pancreatic islet cells from each group. **i** For metabolic flux analyses (Fig. 5i and Supplementary Fig. 2c), 18-week-old NOD pancreatic islet cells from each group were assessed for (Supplementary Fig. 2c) glycolysis after glucose stimulation (*n* = 5) and respiratory capacity (*n* = 5); two-tailed *t*-test; the error bars represent the s.e.m. *P < 0.05, **P < 0.01, ****P < 0.0001. Seahorse measurement was normalized by using total cell number (BioTek Citation 1/5). One-way ANOVA; error bars are the s.e.m. ***P < 0.001, ****P < 0.0001 for comparison between scrambled and MOTS-c. ^†^*P* < 0.05 for comparison between scrambled versus exendin-4 or exendin-4 versus exendin-4 + MOTS-c. **j** NOD-β-cell-derived NIT-1 cells (*n* = 10 cells per slide, *n* = 5 slides per group) overexpressing either empty vector or MOTS-c were analyzed with TEM for mitochondria number and morphology. Two-tailed *t*-test; the error bars are the s.e.m. Scale bars, 1 μm. *P < 0.05, **P < 0.01, ****P < 0.0001 for comparison between scrambled and MOTS-c. ^#^*P* < 0.05, ^###^*P* < 0.001 for comparison between scrambled versus exendin-4. ^††^*P* < 0.01, ^†††^*P* < 0.001, ^††††^*P* < 0.0001 for comparison between scrambled versus MOTS-c + exendin-4.

While T1D is primarily recognized as a T cell-mediated disease, mounting evidence underscores the significance of non-cell-autonomous activities of senescent β-cells in type 1 diabetes and suggests that the targeted elimination of SASP-producing senescent β-cells can prevent autoimmune pancreatic insulitis^2^. In our previous work, we demonstrated that MOTS-c prevents autoimmune diabetes in NOD mice, a model of type 1 diabetes, by specifically targeting spleen and pancreas^7,10^. However, the effects of MOTS-c treatment on NOD β-cells and SASP production remain largely unexplored. Analyses of publicly available T1D-related datasets^34,35^ showed that MOTS-c (mtRNR1) expression levels significantly decrease following treatment with SASP-related cytokines (FDR of 0.032, IFN-γ, IL1β, TNF^14^; FDR of 0.001, IFN-α^15^) (Fig. 2a and Supplementary Table 4). Exendin-4 has been shown to significantly enhance human beta-cell engraftment when used in combination with dual tyrosine-regulated kinase 1A inhibitor, harmine^44^, indicating its potential applicability for patients with T1D. Exendin-4 has been extensively tested in combination with other drugs^44–50^ in mouse models of diabetes. However, its effect on pancreatic islet cell senescence, particularly in combination with MOTS-c, has not been evaluated. Moreover, the mode of action of the exendin-4 differs from that of MOTS-c, necessitating an investigation into their potential synergistic effects. Therefore, we selected exendin-4 as both a positive control and a combination partner for MOTS-c. Given that senescence is a multipathway-driven phenomenon, we employed a combination therapy using MOTS-c and exendin-4 to treat β-cell senescence in NOD mice.

Systemic treatment with MOTS-c (0.5 mg kg^−1^ per day, i.p.)^7^, exendin-4 (2.0 µg kg^−1^, two times a day, i.p.)^23,51^ and a combination of both (Fig. 2b and Supplementary Fig. 2a) was initiated at 7 weeks of age (*n* = 18 per group). The treatments with MOTS-c, exendin-4 and combination treatment reduced blood glucose levels (scrambled: 331.7 ± 200.98 mg dl^−1^, MOTS-c: 200.9 ± 142.25 mg dl^−1^, exendin-4: 127.9 ± 16.82 mg dl^−1^, exendin-4 + MOTS-c: 134.5 ± 14.085 mg dl^−1^) and delayed the onset of diabetes at 18 weeks of age (Fig. 2b); these effects were independent of body weight (Fig. 2c). An IPGTT on 14-week-old NOD mice revealed that MOTS-c, exendin-4 and combined treatment significantly improved glucose tolerance (Fig. 2d). H&E staining of pancreatic tissue indicated that MOTS-c treatment, with or without exendin-4, significantly reduced immune cell infiltration (Fig. 2e).

To explore the effects of MOTS-c treatment on β-cell function and senescence in NOD mice, pancreatic tissues and islets were isolated from 18-week-old NOD mice treated with scrambled peptide, MOTS-c and/or exendin-4. Immunofluorescence staining showed that MOTS-c treatment, whether alone or in combination with exendin-4, reduced the frequency of γ-H2AX expression compared with the scrambled peptide-treated control group in insulin-positive pancreatic islet cells (Fig. 2f and Supplementary Fig. 2b). Further qPCR analysis in NOD pancreatic islet cells showed increased mRNA levels of senescence- and SASP-related genes such as *Igf1r*, *Cdkn1a*, *Cdkn2a*, *Il-1b*, *Il-6* and *Cxcl10* in scrambled peptide-treated group (Fig. 2g). These mRNA expression levels were reduced by MOTS-c treatment and further decreased in the combination treatment group (Fig. 2g). In addition, staining of β-cell senescence and SASP markers, β-gal and p21 (ref. ^13^), in pancreatic islets showed that MOTS-c treatment, with or without exendin-4, decreased β-gal- and p21-double-positive senescent cells in pancreatic islets (Fig. 2h). These findings suggest that MOTS-c treatment prevents pancreatic β-cell senescence in NOD mice and dual treatment with exendin-4 produces synergistic effects in downregulating senescence/SASP-related genes and senescent β-cell subsets.

Senescent cells display mitochondrial dysfunction and altered metabolism in various models of cellular senescence^3,52^. Given that mitochondrial-genome-encoded MOTS-c can regulate metabolism and improve mitochondrial function in various cell types^6–10^, we investigated whether MOTS-c can influence the metabolic function of NOD pancreatic islet cells. MOTS-c treatment, either alone or in combination with exendin-4, upregulated mitochondrial oxidative phosphorlyation (OXPHOS), measured by oxygen consumption rate (OCR) levels—but not glycolysis—as measured by extracellular acidification rate (ECAR) levels, in NOD pancreatic islet cells (Fig. 2i and Supplementary Fig. 2c). Overall, MOTS-c treatment, with or without exendin-4, can prevent β-cell senescence and upregulate mitochondrial OXPHOS to mitigate pancreatic insulitis in NOD mice.

Previous report suggest that MOTS-c can upregulate genes related to mitochondrial biogenesis^53,54^ and our data showed that MOTS-c increased OXPHOS levels in NOD pancreatic islet cells compared with scrambled or exendin-4 treated NOD pancreatic islets (Fig. 2i). To test if the increased mitochondrial OXPHOS is caused by an increased mitochondrial biogenesis, we performed transmission electron microscopy (TEM) in NIT-1 cells, which is an immortalized NOD β-cell line^55^, transfected with either empty or MOTS-c vector. MOTS-c-overexpressing NIT-1 cells showed an increased mitochondrial number and restored curved mitochondrial cristae, contributing to enhanced mitochondrial function^56^, as opposed to the previously observed bulged mitochondria (Fig. 2j). Overall, increased mitochondrial OXPHOS in MOTS-c treated NOD mice might be caused by enhanced mitochondrial morphology, function and increased number of mitochondria.

### Circulating and β-cell MOTS-c levels are low in patients with T2D, and systemic MOTS-c treatment prevents pancreatic islet senescence in S961-treated C57BL/6 mice

**Fig. 3:**
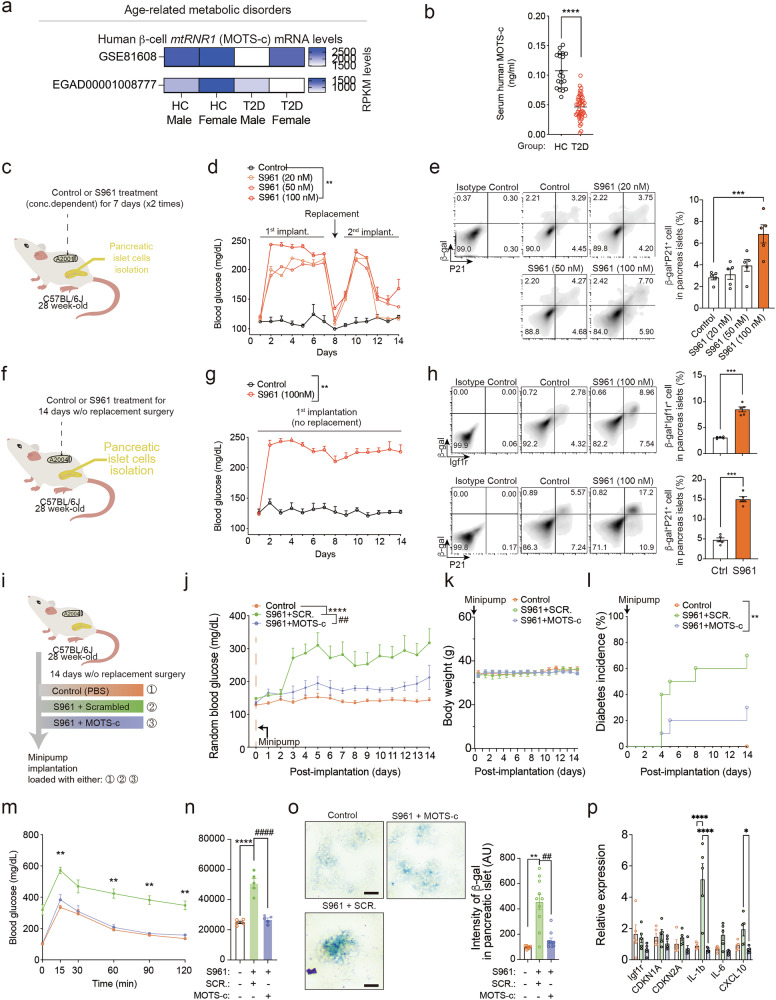
Circulating and β-cell MOTS-c levels are low in patients with T2D, and systemic MOTS-c treatment prevents pancreatic islet senescence in S961-treated C57BL/6 mice. **a** Publicly available datasets from patients with T2D and healthy control patients (GSE81608, EGA00001006273) were analyzed to evaluate *mtRNR1* (MOTS-c) mRNA levels (RPKM levels) (Supplementary Table 4). **b** The serum samples from human patients with T2D (*n* = 45) and healthy controls (*n* = 19) were obtained and analyzed for circulating MOTS-c levels at fasting baseline. Two-tailed *t*-test; the error bars are the s.e.m. ****P < 0.0001. **c**–**e** The insulin-receptor antagonist S961 or PBS (control) (*n* = 5 per group) was loaded in Alzet osmotic pump 2001, which was then implanted in 28-week-old C57BL/6 mice for 1 week. **d** After a week, replacement surgery was performed to change the osmotic pump for the second week; blood glucose levels were monitored throughout the 14-day period and after sacrifice, (**e**) β-gal^+^P21^+^ population in pancreatic islets (*n* = 5 per group) was analyzed. Two-way ANOVA; the error bars are the s.e.m. ***P < 0.001. **f** The insulin-receptor antagonist S961 or PBS (control) (*n* = 5 per group) was loaded in Alzet osmotic pump 2004, which was then implanted in 28-week-old C57BL/6 mice for 2 weeks without further replacement surgery. **g**,**h** The blood glucose levels were (**g**) monitored for the entire 14 days and after sacrifice, and (**h**) β-gal^+^Igf1r^+^ and β-gal^+^P21^+^ population in pancreatic islets (*n* = 5 per group) were analyzed. Two-way ANOVA; the error bars are the s.e.m. ***P < 0.001. **i** The insulin-receptor antagonist S961, along with either MOTS-c or scrambled peptide, was loaded into Alzet osmotic pump 2004 and implanted in 28-week-old C57BL/6 mice for 2 weeks. **j**–**o** (**j**) Random blood glucose, (**k**) body weight, (**l**) diabetes incidence (**m**), IPGTT, (**n**) area under the curve (AUC), (**o**) and β-gal staining was performed after 2 weeks of S961 treatment. The pancreatic sections were prepared as described; representative data are from ten pancreas sections for each group (*n* = 10 per group). Scale bars, 50 μm. One-way, two-way or repeated measures two-way ANOVA; the error bars are the s.e.m. **P < 0.01, ****P < 0.0001 for comparison between control versus S961 + scrambled. ^##^*P* < 0.01, ^####^*P* < 0.0001 for comparison between S961 + scrambled versus S961 + MOTS-c. **p** The pancreatic islet cells were isolated from 28-week-old C57BL/6 mice treated in vivo with either PBS, S961 + scrambed or S961 + MOTS-c for 2 weeks. The pancreatic islet cells were then incubated in culture media to obtain ‘conditioned’ media. Fresh pancreatic islet cells were isolated from different mice and incubated with the ‘conditioned’ media for 24 h. Finally, the cells in conditioned media were collected and analyzed by qPCR on *Cdkn1a*, *Cdkn2a*, *IL-1b* and *Cxcl10*. Two-way ANOVA; the error bars are the s.e.m. *P < 0.05, **P < 0.01, ****P < 0.0001. **i** A diagram depicting the possible role of MOTS-c in β-cell senescence and SASP.

Mitochondrial functions, such as mitochondrial metabolism, ATP or the production of mitochondrial-derived peptides, decline with aging in mice and in patients with T2D^6,8,9,57–69^. By analyzing two publicly available datasets, we found that T2D male and female β-cells exhibited lower mRNA expression levels of MOTS-c (*mtRNR1*) compared with the healthy controls (Fig. 3a). To confirm this, we obtained serum samples from healthy controls (*n* = 19, age 29.1 ± 6.8 years, female 26%) and patients with T2D (*n* = 45, age 64.7 ± 7.9 years, female 51%) to measure circulating MOTS-c levels (Fig. 3b and Supplementary Fig. 1). The circulating levels of MOTS-c was significantly lower in patients with T2D compared with healthy controls (Fig. 3b). However, given that the T2D group was older, it remains unclear whether the observed difference in MOTS-c levels is primarily attributable to aging, diabetes or a combination of both.

Systemic treatment with the insulin-receptor antagonist, S961, can induce β-cell-specific senescence and hyperglycemia in 28-week-old C57BL/6J mice^1,12^. However, the current method for using the Alzet osmotic pump 2001, loaded with 20 nM of S961, requires invasive surgical procedures to replace the pump every week^1,12^ (Fig. 3c). To address this limitation, we used the Alzet osmotic pump 2004, loaded with 100 nM of S961, which lasts for the entire 2-week experiment (Fig. 3f). A comparison between different concentrations of S961 delivered by the Alzet 2001 pump showed that 100 nM of S961 induced both hyperglycemia and a higher frequency of β-gal and P21 double-positive pancreatic islet cells compared to the 20 or 50 nM loaded groups (Fig. 3d,e). We then used 100 nM S961 administered through the Alzet osmotic pump 2004 (Fig. 3f) with a single surgery. This resulted in a more consistent level of blood glucose levels and higher β-gal and P21 double-positive senescent pancreatic islet cell frequency compared to the dual-surgery model (Fig. 3d–h).

After establishing the single-surgery mouse model, we tested whether MOTS-c can prevent β-cell-specific senescence (S961)-induced hyperglycemia. To test this, MOTS-c or scrambled peptide were treated in 28-week-old C57BL/6 mice for 2 weeks with S961 (Fig. 3i). S961 and scrambled peptide treatments increased random blood glucose levels, while MOTS-c treatment reduced blood glucose elevation and lowered diabetes incidence (70% versus 30%) induced by S961, without body weight change (Fig. 3j–l). IPGTT after 2 weeks of treatment revealed that MOTS-c administration significantly improved glucose tolerance (Fig. 3m,n). To confirm the anti-senescence effect of MOTS-c, we analyzed pancreatic islets for senescence by staining for β-gal activity. MOTS-c treatment was associated with lower β-gal-stained cells compared with those treated with scrambled peptide, suggesting that MOTS-c can prevent pancreatic islet cell senescence induced by S961 (Fig. 3o). Furthermore, MOTS-c lowered SASP-related genes such as *Il1b* and *Cxcl10* suggesting a cell-autonomous regulation by MOTS-c in pancreatic islet cells (Fig. 3p).

### MOTS-c regulates genes and metabolites associated with aspartate–glutamate transport to prevent senescence in pancreatic islet and Min6 cells

**Fig. 4:**
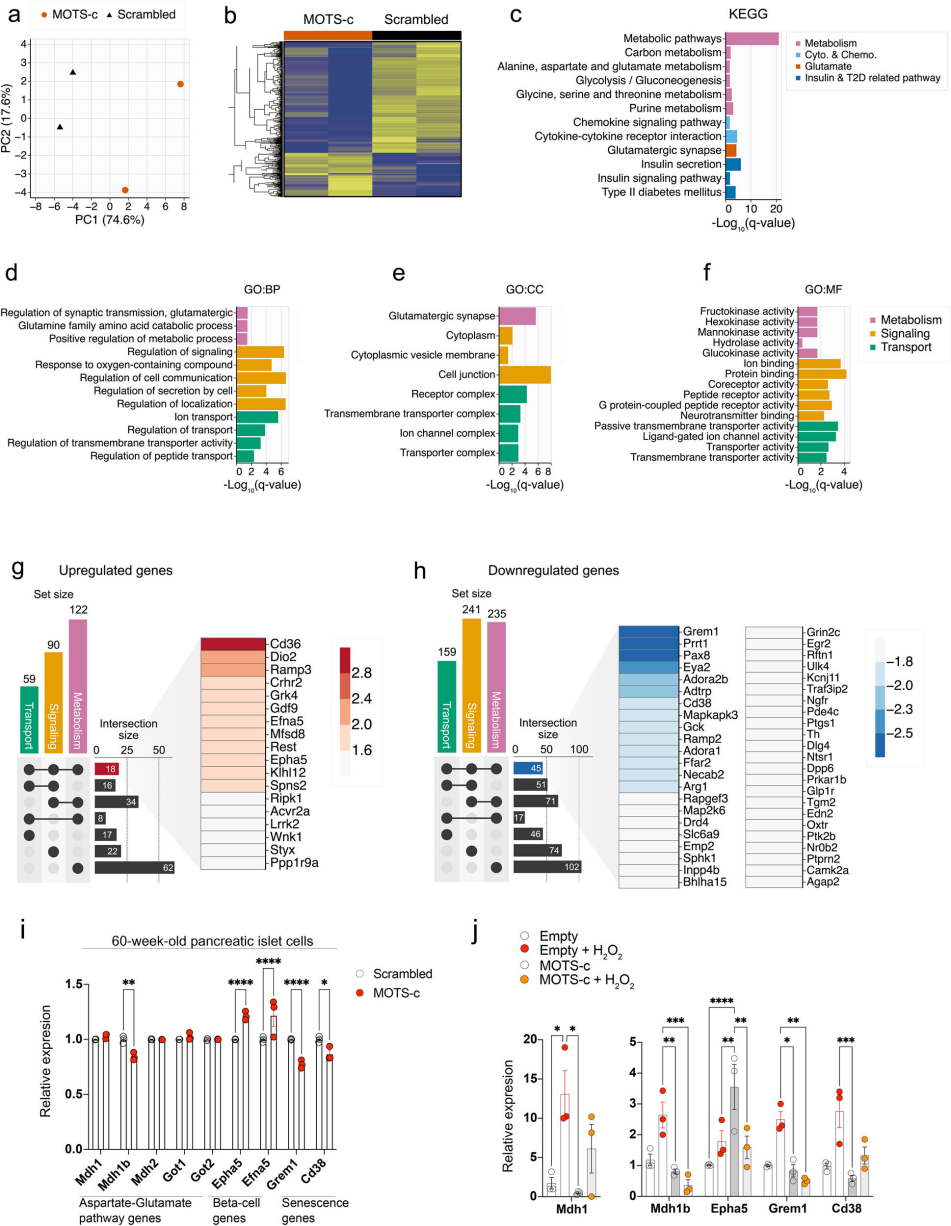
MOTS-c regulates genes associated with aspartate–glutamate transport to prevent senescence in pancreatic islet and Min6 cells. **a**,**b** MOTS-c or scrambled ex vivo treatment (10 μM, 24 h) was applied to pancreatic islet cells isolated from four littermates of 60-week-old C57BL/6 mice (*n* = 2 per group) to analyze transcriptional changes; these changes were assessed using a PCA plot analyzed by using the scikit-learn Python package (**a**) and a hierarchical heat map (**b**). **c**–**f** KEGG and GO analyses (adjusted *P* value <0.05) indicated that the affected genes are associated with metabolism, cellular communication and signaling and transport (**c**); analysis included: Gene Ontology: biological process (GO: BP) (**d**) Gene Ontology: cellular components (GO: CC) (**e**) Gene Ontology: molecular function (GO: MF) (**f**) were analyzed. **g**,**h** The upset plots were used to identify intersecting sets, which are commonly shared genes related to metabolism (pink), signaling (orange) and transport (green); these commonly shared genes, categorized as either upregulated (**g**) or downregulated (**h**) (blue), were displayed in a heat map. **i**, MOTS-c or scrambled ex vivo treatment (10 μM, 24 h) was applied to pancreatic islet cells isolated from littermates of 60-week-old C57BL/6 mice (*n* = 3 per group). **j** pLJM1-MOTS-c or pLJM1-empty vectors were overexpressed in Min6 cells. Then, cells were treated with or without hydrogen peroxide (200 μM, 24 h) for senescence induction. Subsequently, the expression of genes involved in aspartate–glutamate pathway (*Mdh1*, *Mdh1b*, *Mdh2*, *Got1* and *Got2*), EphA5-ephrina5 genes and senescence-related genes (*Cd38* and *Grem1*) were analyzed in both sets.

**Fig. 5:**
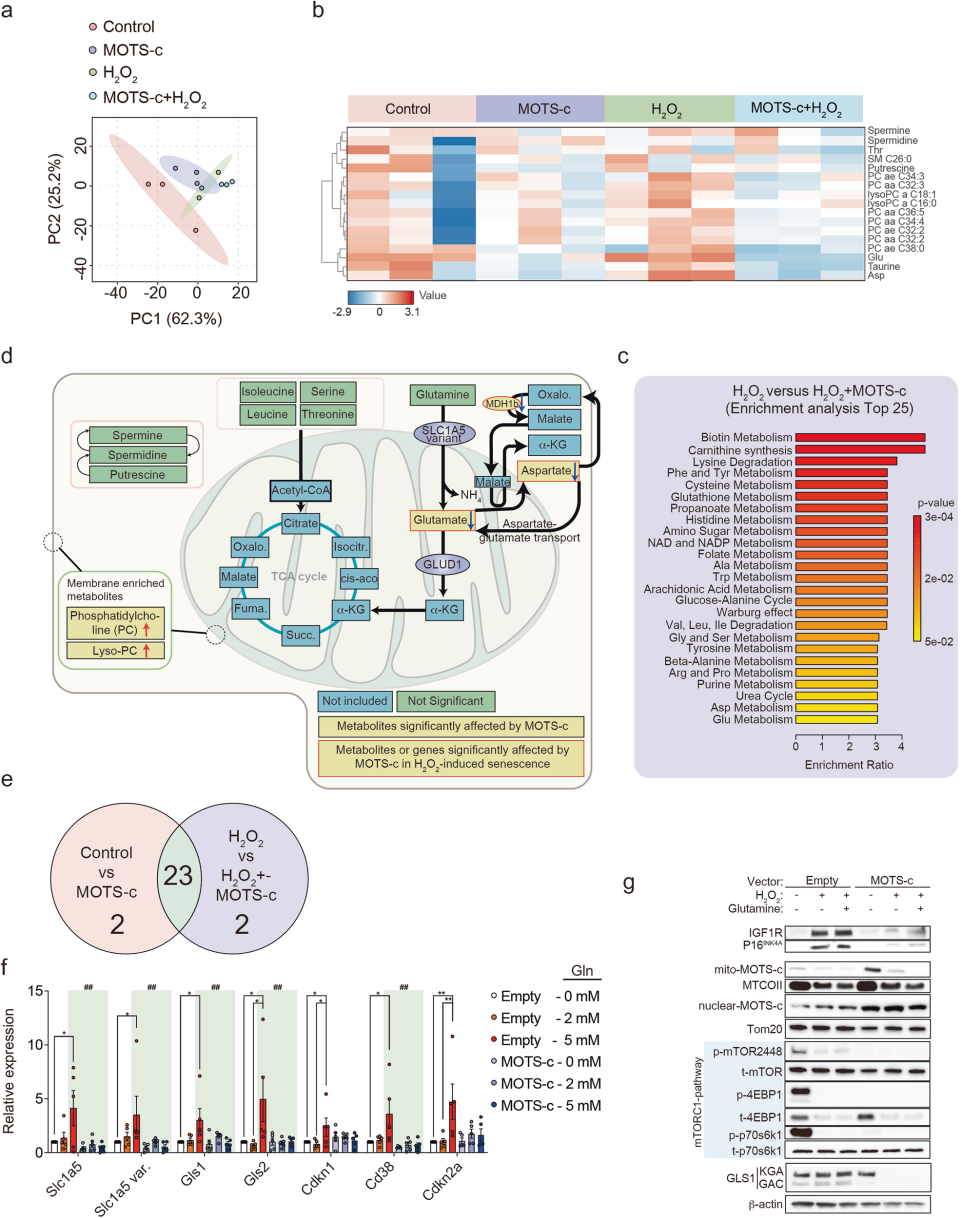
MOTS-c regulates metabolites associated with glutamate transport to prevent senescence in pancreatic islet cell and Min6 cells. **a**,**b** Treatment with MOTS-c or scrambled control (10 μM, 24 h) and hydrogen peroxide (H_2_O_2_, 200 μM, 24 h) in pancreatic islet cells (pooled from two mice per sample) isolated from littermates of 60-week-old C57BL/6 mice (*n* = 3 per sample) led to metabolic changes, as shown in the PCA graph (**a**) and the heat map (**b**). **c** Enrichment analyses of metabolites were performed for control versus MOTS-c and for H_2_O_2_ versus H_2_O_2_ + MOTS-c. **d** A diagram depicting the enriched genes and metabolites analyzed in pancreatic islet cells treated with or without MOTS-c and H_2_O_2_ (200 μM, 24 h). **e** A Venn diagram analysis was performed to find shared pathways by comparing these two enrichment analyses. **f** Min6 cells overexpressing either empty vector or MOTS-c were treated with glutamine and the expression of genes *Slc1a5*, *Slc1a5* variant, *Gls1/2* and *Cd38*, and *Cdkn1a* and *Cdkn2a* were assessed. Two-way ANOVA; the error bars are the s.e.m. *P < 0.05, **P < 0.01 for difference between empty-vector transfected; ^##^*P* < 0.01 for difference between empty-vector transfected treated with 5 mM glutamine and MOTS-c transfected treated with 5 mM glutamine. **g** Min6 cells overexpressing either an empty vector or MOTS-c were analyzed for protein levels of IGF1R, P16, mito-MOTS-c, nuclear MOTS-c- and mTORC1-related molecules and Gls1 in the presence or absence of glutamine (5 mM), H_2_O_2_ (200 μM, 24 h) or both.

MOTS-c treatment in HEK293 cells showed increased metabolic signaling, fatty acid, nucleotide and ubiquitin signaling but decreased ribosome component, inflammation and cytokine-related pathways^8^. To understand the mechanism underlying the role of MOTS-c in protecting pancreatic islets from senescence, we performed gene microarray analyses in pancreatic islet cells treated with either MOTS-c or a scrambled peptide; the cells were isolated from four littermates of 60-week-old C57BL/6 mice (*n* = 2 per group) (Fig. 4e). Principal component analysis (PCA) revealed a clear shift in gene expression between scrambled- and MOTS-c treated groups (Fig. 4a). Hierarchical clustering and enrichment analyses further accentuated the shift in gene expression pattern by MOTS-c treatment in pancreatic islet cells (Fig. 4b). The analyses using KEGG database suggest that these genes were involved in diabetes-related, insulin signaling-related, insulin secretion-related, cytokine and chemokine-related and asparate/glutamate-related pathways (Fig. 4c). Using GO database, many MOTS-c-dependent genes that were differentially downregulated were strongly associated with metabolism-related, transport-related and signaling-related pathways (Fig. 4d–f). To further elucidate the commonly shared genes potentially involved in metabolism, transport and signaling pathways, we generated UpSet plots (Fig. 4g,h). We identified 18 genes upregulated and 45 genes downregulated in a MOTS-c-dependent manner (Fig. 4g,h). Notably, among the genes upregulated by MOTS-c, several are closely associated with fatty acid transport (*Cd36*)^70^ and with beta-cell communication and insulin secretion (*Efna5* and *Epha5*)^22^ (Fig. 4g). By contrast, genes downregulated by MOTS-c included those strongly linked to cellular senescence such as *Grem1* and *Cd38* (refs. ^18,19,71–75^) (Fig. 4h and Supplementary Fig. 3a), suggesting a potential anti-senescent role for MOTS-c. To validate the effect of MOTS-c on the aspartate–glutamate pathway and senescence-related processes, we isolated pancreatic islet cells from 60-week-old male mice and treated them ex vivo with either MOTS-c or scrambled peptides for 24 h. We then performed qPCR analysis on genes related to the aspartate–glutamate pathway (*Mdh1*, *Mdh1b*, *Mdh2*, *Got1* and *Got2*), beta-cell communication and insulin secretion (*Epha5* and *Efna5*) and senescence (*Grem1* and *Cd38*). MOTS-c treatment significantly reduced the expression of *Mdh1b*, *Grem1* and *Cd38* in 60-week-old pancreatic islet cells (Fig. 4i), while the expression levels of *Mdh1*, *Mdh2*, *Got1* nor *Got2* remained unchanged. Conversely, *Epha5* and *Efna5* were upregulated following MOTS-c treatment, indicating enhanced beta-cell communication and insulin production (Fig. 4i). To further substantiate these findings, we overexpressed MOTS-c or empty-vectors in Min6 cells (Fig. 5g). The cells were subsequently treated with H_2_O_2_ to induce senescence and qPCR analysis was performed. Expression of *Mdh2*, *Got1*, *Got2* and *Efna5* was undetected in Min6 cells (data not shown). MOTS-c overexpression significantly reduced the expression of *Mdh1b*, *Grem1* and *Cd38* (Fig. 4j). In addition, *Epha5* expression was increased by MOTS-c overexpression but was suppressed by H_2_O_2_ treatment (Fig. 4j). Collectively, these data suggest that MOTS-c modulates gene expression by downregulating senescence-related genes (*Grem1* and *Cd38*) and the aspartate/glutamate-related *Mdh1b*, while upregulating *Epha5*, a key gene involved in β-cell function.

MOTS-c regulates metabolism across various tissues and animal models^6–10,76–87^. However, whether MOTS-c can influence pancreatic islet metabolism in aged mice remains unclear. To gain a metabolic overview of pancreatic islet cells treated with MOTS-c, we performed targeted metabolomics analysis on pancreatic islet cells, which were isolated from 60-week-old C57BL/6 littermate mice and ex vivo treated with MOTS-c and/or H_2_O_2_. Previous report suggests that H_2_O_2_ treatment in pancreatic β-cells induce senescence^1^. PCA revealed a clear shift in metabolite profiles between control, H_2_O_2_, MOTS-c and MOTS-c + H_2_O_2_ treated groups derived from multiple components (Fig. 5a). Pathways involving glutamate, aspartate and lipids (phosphatidylcholines (PC); lyso-PC) were substantially enriched by MOTS-c (Fig. 5b–d and Supplementary Fig. 3b). Increased lipids and urea-related metabolism correlate with previous data from MOTS-c-treated HEK293 cells^8^ and liver tissues with non-alcoholic steatosis hepatitis^88^. Furthermore, 23 metabolic pathways were commonly enriched in two different analyses (control versus MOTS-c, H_2_O_2_ versus H_2_O_2_ and MOTS-c) (Fig. 5e and Supplementary Fig. 3c). Among the metabolites related to these 23 pathways, urea, glutamate (Glu) and aspartate (Asp) levels were downregulated by MOTS-c treatment and PCs (PC acyl-acyl (aa) C34:1, PC acyl-acyl (aa) C38:4, PC acyl-ether (ae) C38:5, lysoPC acyl (a) C16:0) were upregulated by MOTS-c (Supplementary Fig. 4a–d). Upon senescence induction using H_2_O_2_, glutamate and aspartate were downregulated by MOTS-c treatment (Fig. 5d and Supplementary Fig. 4a–d). To further investigate how MOTS-c regulates aspartate–glutamate metabolism, Min6 cells were treated with MOTS-c and/or H_2_O_2_, and then, the metabolites were analyzed using LC–MS/MS. Consistent with observations in aged pancreatic islet cells, MOTS-c treatment in H_2_O_2_-induced senescent Min6 cells appeared to further reduce glutamate and malate levels (Supplementary Fig. 5c). Considering aspartate is mainly synthesized in the mitochondrial matrix through tricarboxylic acid cyle^89^, MOTS-c might be involved in regulating mitochondrial aspartate–glutamate metabolism.

Previously, we have shown that intracellular MOTS-c levels can be lowered in an mTORC1-dependent manner in Jurkat cells^7^. Furthermore, Lee et al. have shown that AMPK activation by AICAR treatment increases MOTS-c levels in skeletal muscle and HEK293 cells^6,8^, and AMPK inhibitor by compound C treatment decreases MOTS-c levels^6^. To determine which metabolic regulator, AMPK or mTORC1, affect MOTS-c expression levels in Min6 β-cell lines, we treated the Min6 cells with mTORC1 activator (MHY1485), its inhibitor (rapamycin) and the AMPK activator (AICAR) and its inhibitor (compound C) (Supplementary Fig. 5a). AICAR increased phosphorylated AMPK and MOTS-c levels without altering p-mTOR (2448) levels, while compound C decreased the phosphorylation of AMPK and p-mTOR (2448) and total mTOR protein (Supplementary Fig. 5a). MHY1485 increased the phosphorylation of p-mTOR (2448) and decreased MOTS-c levels (Supplementary Fig. 5a). Rapamycin treatment decreased the phosphorylation of p-mTOR (2448) and increased MOTS-c levels, as reported^7^. Considering the activation and inhibition of p-mTOR (2448) showed higher consistency compared with AMPK activator and inhibitor, we focused on mTORC1 pathways.

Glutamine, IGF1 and CD3 and CD28 antibodies treatment lower MOTS-c levels in Jurkat cells^7^, and MOTS-c overexpression increase NAD^+^ levels and decrease glutamine levels in HEK293 cells^8^. Glutaminolysis produces glutamate and ammonium ions, both of which are essential for the survival of senescent cells^90–92^. To further understand the relationship between MOTS-c and β-cell senescence, glutamine was treated in either empty or MOTS-c-overexpressed Min6 cells cultured in glutamine-depleted media. Glutamine increased the mRNA levels of both plasma membrane and mitochondrial glutamine-related transporters (*Slc1a5* and *Slc1a5* variant), glutaminase (*Gls1* and *Gls2*), NAD^+^-consuming enzyme (*Cd38*) and senescence-related genes (*Cdkn1a* and *Cdkn2a*) in empty-transfected Min6 cells (Fig. 5f). MOTS-c overexpression in Min6 cells were able to lower these glutaminolysis-related genes (Fig. 5f). To further assess whether MOTS-c can prevent either H_2_O_2_ alone or both H_2_O_2_- and glutaminolysis-induced β-cell senescence, Min6 cells overexpressed with empty or MOTS-c vectors were treated with H_2_O_2_ or H_2_O_2_ and glutamine. MOTS-c overexpression lowered senescence markers such as Gls1, Igf1r and P16^INK4a^ expression levels induced by H_2_O_2_ alone or H_2_O_2_ and glutamine (Fig. 5g). MOTS-c is coordinated by both mTORC1 and AMPK activities and utilizes Nrf2 to recognize and bind to antioxidant response elements^6,7,76^. In our data, MOTS-c treatment lowered mTORC1-related genes such as *Cd38*, *Camk2a*, *Map2k6*, *Gck* and *Ulk4*^71,93–95^ (Fig. 4h). It has been reported that H_2_O_2_ treatment in β-cells interrupt mTORC1 signaling leads to loss of β-cell identity, prevents insulin production and induces senescence (γ-H2AX)^96–99^. MOTS-c overexpression in Min6 cells increased both nuclear and mitochondrial MOTS-c under control conditions. However, under H_2_O_2_-treated conditions, nuclear MOTS-c increased to a greater extent than mitochondrial MOTS-c, suggesting a mitochondrial-to-nuclear translocation of MOTS-c (Fig. 5g). Simultaneously, augmentation of nuclear MOTS-c inhibited mTORC1 activity, determined by reduced phosphorylation of 4EBP-1, P70S6K1 and mTOR (2448) (Fig. 5g). These results suggest that MOTS-c could reduce senescence markers (IGF1R, P16^INK4a^ GLS1) in an mTORC1-dependent pathway.

### MOTS-c treatment prevents senescence in an mTORC1-dependent manner in pancreatic islet and Min6 cells

**Fig. 6:**
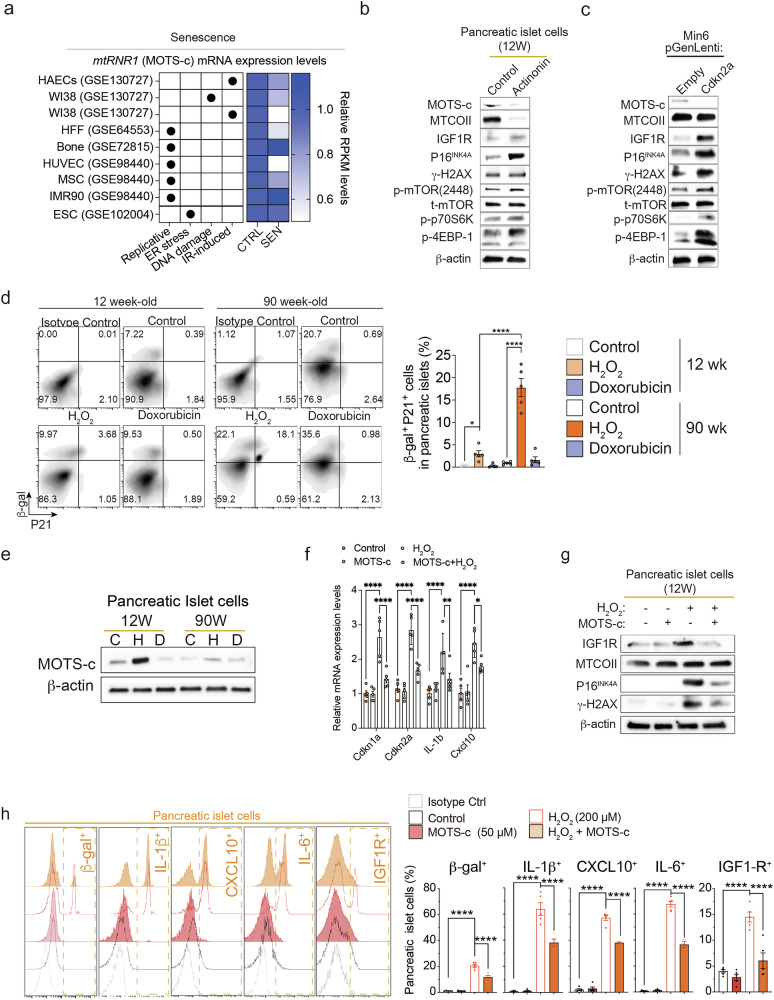
MOTS-c treatment prevents senescence in an mTORC1-dependent manner in pancreatic islet and Min6 cells. **a** Publicly available datasets (GSE137027, GSE64553, GSE72815, GSE98440 and GSE102004) were analyzed for *mtRNR1* (MOTS-c) mRNA expression levels (Supplementary Table 4). **b** To explore the underlying mechanism of MOTS-c regulation in senescence, actinonin (50 μM, 24 h) was used to specifically deplete mtDNA in pancreatic islet cells isolated from 12-week-old C57BL/6 mice. **c**, Min6 cells were transfected with pGenLenti-empty or pGenLenti-Cdkn2a vectors. In **b** and **c** the senescence markers (Igf1r, P16INK4a and γ-H2AX) and mTORC1 pathway-related (p-mTOR-2448, p-p70S6K and p-4EBP-1) proteins were analyzed. The pancreatic islet cells from 12- and 90-week-old mice were treated with either hydrogen peroxide (200 μM, 24 h) and doxorubicin (200 nM, 24 h). **d** The β-gal^+^p21^+^ population in pancreatic islets were analyzed. Two-way ANOVA; the error bars are the s.e.m. *P < 0.05, ****P < 0.0001 for comparison. **e** Hydrogen peroxide and doxorubicin were treated in pancreatic islet cells isolated from 12- or 90-week-old mice to analyze MOTS-c levels. All western blot data are representative of at least three independent experiments. **f** Treatment with MOTS-c (10 μM, 24 h), with or without H_2_O_2_ (200 μM, 24 h), in pancreatic islet cells isolated from 12-week-old C57BL/6 mice prevented senescence markers, including *Cdkn1a*, *Cdkn2a*, *Cxcl10* and *Il-1b* mRNA levels. Two-way ANOVA; the error bars are the s.e.m. *P < 0.05, **P < 0.01, ****P < 0.0001 for comparison. **g** Pancreatic islet cells isolated from 12-week-old C57BL/6 mice were treated with H_2_O_2_ to analyze protein expression levels of γ-H2AX and P16^INK4A^. Housekeeping mitochondrial and cytoplasmic proteins (MTCOII and β-actin) were confirmed by western blot. **h** Treatment with MOTS-c (10 μM, 24 h) in the presence or absence of H_2_O_2_ (200 μM, 24 h) in pancreatic islet cells isolated from 12-week-old C57BL/6 mice (*n* = 5 per group) was followed by staining and analysis for β-gal, IL-1β, Cxcl10, IL-6 and Igf1r using flow cytometry. Two-way ANOVA; the error bars are the s.e.m. ****P < 0.0001 for comparison.

Using publicly available datasets^38–42^, we observed that *mtRNR1* (MOTS-c) mRNA expression levels are decreased by senescence in different types of cell (Fig. 6a). To understand the relationship between the loss of MOTS-c expression levels and senescence, actinonin was employed to deplete mitochondrial RNA^6–8^. Actinonin treatment depleted MOTS-c expression in pancreatic islet cells isolated from 12-week-old mice (Fig. 6b). Upon depletion of MOTS-c expression in pancreatic islet cells, senescence markers such as Igf1r, P16^INK4A^ and γ-H2AX were upregulated and activated mTORC1-signaling proteins such as p-mTOR (2448), p-p70S7K and p-4EBP-1 (Fig. 6b). Similarly, overexpressing P16^INK4A^ (*Cdkn2a*) in Min6 cells lowered MOTS-c expression while increasing senescence markers and activating mTORC1-signaling pathways compared with empty-vector transfected Min6 cells (Fig. 6c).

Oxidative stress induces β-cell senescence and SASP production, and alters MOTS-c expression levels in different cell types^1,6,12^. Since chronological aging leads to a decrease in MOTS-c levels in pancreatic islet cells (Fig. 1d), we exposed pancreatic islet cells isolated from 12- and 90-week-old mice to H_2_O_2_ (200 μM, 24 h) or doxorubicin (200 nM, 24 h) to mimic chronological aging in vitro. H_2_O_2_ treatment increased the β-gal^+^P21^+^ population in pancreatic islet isolated from 12-week-old C57BL/6 mice and further accentuated in pancreatic islet isolated from 90-week-old C57BL/6 mice (Fig. 6d). Interestingly, MOTS-c levels were lower in H_2_O_2_-treated 90-week-old pancreatic islets compared with 12-week-old mice (Fig. 6e). This suggests that aged pancreatic islet cells were unable to produce endogenous MOTS-c in response to H_2_O_2_-induced senescence (Fig. 6e). Conversely, exogenous MOTS-c treatment lowered both mRNA and protein levels of senescence markers (γ-H2AX, Igf1r and P16^INK4A^) and SASP markers (*Il-1b*, *Cxcl10*) in pancreatic islet cells isolated from 12-week-old mice (Fig. 6f,g).

To assess whether MOTS-c can regulate SASP (IL-1β, CXCL10, IL-6 and TNF), MOTS-c was treated with or without H_2_O_2_ in pancreatic islet cells isolated from 12-week-old C57BL/6 mice. Concentration-dependent MOTS-c treatment did not alter the production of IL-1β, CXCL10, IL-6 and TNF in pancreatic islet cells (Supplementary Fig. 5b). Senescence induction by H_2_O_2_ treatment induced the increased production of IL-1β, CXCL10 and IL-6 but not TNF (Supplementary Fig. 5b). In the presence of H_2_O_2_, concentration-dependent MOTS-c treatment reduced the production of IL-1β, CXCL10 and IL-6 but not TNF (Supplementary Fig. 5b). Finally, we showed that both senescence (β-gal and IGF1R) and SASP markers (IL-1β, CXCL10 and IL-6) were lowered by MOTS-c (Fig. 6h).

## Discussion

Over the past decade, both preclinical and clinical studies have highlighted the efficacy of drugs targeting cellular senescence and their potential applications in diabetes. Senescent β-cells have been detected in rodent models of T1D and T2D and senolytic approaches in islets have shown to improve glucose tolerance^1,2,12,24^. However, β-cell-specific mitochondrial markers for phenotyping or distinguishing senescent β-cells have not yet been studied. Moreover, the therapeutic potential of mitochondrial peptides on β-cell senescence has not been explored. Our work demonstrates that MOTS-c can modify unique senescence-associated transcriptional and metabolic profiles in pancreatic β-cells. Systemic MOTS-c treatment prevents β-cell senescence, a key inflammation process, in rodent models of both type 1 and type 2 diabetes, highlighting its potential as a senotherapeutic drug.

In this study, we evaluated the role of MOTS-c in regulating aging or senescence in murine β-cells. By performing microarray gene expression analysis in β-cells isolated from 60-week-old mice, we found that MOTS-c downregulates genes related to mTOR pathway (*Cd38*, *Camk2a*, *Map2k6*, *Gck*, and *Ulk4*)^71,93–95^ and serine/threonine protein kinase (*Ripk1*, *Styx*, *Wnk1*, *Mapkapk3* and *Ptk2b*), which regulates protein phosphorylation (Fig. 4h). In Min6 cells, overexpression of MOTS-c downregulates basal levels and phosphorylation of mTORC1-related pathways in β-cells (p-mTOR2448, p-4EBP-1 and p-P70S6K1), which is consistent with our previous report in T cells^7^ (Fig. 5g). mTORC1 signaling can activate NAD^+^-consuming enzyme Cd38, which serves as a senescence marker. *Cd38*-positive senescent cells have declined NAD^+^ levels and mitochondrial dysfunction^18,19^ and Cd38-knockout mice exhibit higher NAD^+^ levels and are safeguarded against obesity and metabolic syndrome^72^. Given that MOTS-c plays a potential role in increasing NAD^+^ levels^8^ and Cd38 is expressed in β-cells (Fig. 4g), the reduction of *Cd38* mRNA by MOTS-c in β-cells indicate the potential mitochondrial-peptidyl regulation of NAD^+^-related metabolites in β-cell senescence and aging.

For metabolomics, we performed 188 targeted metabolite LC–MS/MS analysis, but 92 metabolites were not detected or partially detected in some groups. This limitation might have reduced the total number of significant associations in the metabolite analysis in aged pancreatic β-cells. Nevertheless, the overlapping metabolic pathways in two senescence conditions jointly suggested that MOTS-c treatment triggered the reduction of pathways related with glutamate and aspartate in β-cells (Fig. 5). In line with this finding, a recent study has shown that MOTS-c also regulates alanine, aspartate and glutamate metabolism in the liver^88^. As senescent cells rely on glutaminolysis to neutralize their acidic intracellular pH by providing ammonium ions^90^, reduced signature of glutamate-related pathways support the anti-senescent properties of MOTS-c in β-cells. This observation was further supported in glutamine-treated Min6 cells as MOTS-c overexpressed cells had lower expression of glutaminolysis-related genes such as *Slc1a5*, *Slc1a5* variant, *Gls1*, *Gls2* and *Cd38* (Fig. 5f). We further investigated whether this transcriptional regulation is related with mTORC1. Upon H_2_O_2_ treatment, nuclear translocated MOTS-c might be involved in downregulation of serine/threonine kinase (Fig. 4h), which subsequently reduced the phosphorylation of 4E-BP1, P70S6K1 and mTOR^99,100^. In addition, mTORC1 stimulates glutamine metabolism and vice-versa by regulating Gls1^101–103^. The reduction of mTORC1 might also led to the decreased glutamine metabolism and Gls1 expression level.

The relationship between cellular senescence and T1D involves a nuanced interplay between immune responses, β-cell functionality and cellular senescence^2^. Recent research indicates that β-cell senescence also plays a significant role in T1D progression^2^. Senescent β-cell subsets can exacerbate immune responses and produce a SASP, promoting inflammation and further senescence^2^. This creates a feedback loop of inflammation and β-cell dysfunction. Circulating MOTS-c levels were lower in T1D patients compared with healthy controls^7^. Furthermore, systemic MOTS-c treatment can downregulate proinflammatory IFN-γ and IL-17A cytokine production in NOD pancreas tissues suggesting the noncell-autonomous regulation by MOTS-c^7^. Understanding noncell-autonomous regulation of MOTS-c in pancreatic β-cell may reveal new therapeutic targets and enhance our comprehension of T1D pathogenesis. There is increasing evidence of therapeutic efficacy regarding mitochondrial peptides in T1D^7,10^. Humanin, a mitochondrial peptide encoded within the mitochondrial 16S rRNA, was shown to decrease TNF and IFNγ-induced β-cell apoptosis measured by caspase 3/7 activity in NOD mice^104^. We have previously shown that systemic treatment of MOTS-c can prevent pancreatic islet destruction in NOD mice through the regulation of mTORC1 signaling in T cells^7^. However, the role of MOTS-c in β-cell of NOD mice was largely unknown. MOTS-c treatment alone was able to improve glucose tolerance in NOD mice, to a level similar to that of exendin-4 treatment (Fig. 2b). However, MOTS-c treatment—but not exendin-4—was significantly associated with reduced γ-H2AX^positive^ cell proportion and increased OXPHOS activity in pancreatic β-cells (Fig. 2i and Supplementary Fig. 2c). These results suggest a possible superior anti-senescence effect of MOTS-c over exendin-4 in β-cells, with similar glucose-lowering efficacy. Interestingly, cotreatment of MOTS-c and exendin-4 had an enhanced effect than MOTS-c alone in lowering the expression levels of senescence related genes (*Cd38*, *Cdkn1a*, *Cdkn2a*, *Il1b*, *Il6* and *Cxcl10*) in NOD pancreatic β-cells (Fig. 2g). In sum, these data suggest that cotreatment of exendin-4 and MOTS-c might be beneficial for ameliorating senescent β-cells in T1D.

Epidemiological studies in T2D have found decreased circulating levels of mitochondrial-derived peptides such as MOTS-c in people with old age and obesity^10^. Several mtDNA single nucleotide polymorphisms have been reported to be associated with the risk of T2D in both European and East Asians, underscoring the role of mitochondria in the pathogenesis of T2D^66,105^. Moreover, MOTS-c treatment reversed diet-induced obesity and age-dependent insulin resistance in mice^8^, while treatment with K14Q-MOTS-c could not^106^. Furthermore, systemic MOTS-c treatment protected pancreatic islets from T cell-mediated infiltration in autoimmune NOD mice^7,10^ and STZ-induced β-cell apoptosis in gestational diabetic C57BL/6J mice^87^. Considering mitochondrial dysfunction in pancreatic β-cells of T1D and T2D^7,10^ and the reduction of MOTS-c with age^8–10^, the insulin-sensitizing peptide MOTS-c requires further assessment in regulating β-cell senescence. MOTS-c also has salutary effects in insulin resistance and T2D. MOTS-c has been shown to regulate metabolic homeostasis by enhancing skeletal muscle insulin sensitivity^8,68^. An mtDNA polymorphism (m.1382A > C), which leads to a K14Q amino acid change in MOTS-c, was associated with increased susceptibility to T2D in Korean men^106^. In addition, MOTS-c treatment reversed STZ-induced β-cell apoptosis, diet-induced obesity and age-dependent insulin resistance in mice^8,87^. We further evaluated the protective effect of MOTS-c in pancreatic β-cells of S961-treated insulin-resistant mice. MOTS-c treatment delayed diabetes onset, improved glucose intolerance and reduced β-gal^positive^ senescent β-cells in insulin-resistant mice. Interestingly, MOTS-c whole-body administration has shown significant effect in regulating SASP components such as Il-1β and Cxcl10. The cytokine regulation by MOTS-c was also observed in others^7,107–110^, suggesting a possibility as a paracrine mediator and has a potent role as a senomorphic drug for insulin resistance and T2D. Considering that MOTS-c can downregulate the production of inflammatory cytokines in multiple models, MOTS-c may act as a regulator of noncell-autonomous signals that could affect surrounding or remote cells and organs. It has been previously shown that MOTS-c treatment reduces inflammatory cytokine production in NOD mice^7,10^, which might be largely contributed by its effect on β-cells (Figs. 1c,e, 2g, 3p and 6f,h). Further research is required to understand the underlying mechanism of mitochondrial regulation in cytokine production.

In our previous report on T1D and MOTS-c, we observed that circulating MOTS-c levels in human patients with T1D are lower than those in healthy controls^7^. Analysis of publicly available datasets for *mtRNR1* (MOTS-c) mRNA levels in pancreatic islet cells or EndoC-HB1 cells treated with inflammatory cytokines showed that MOTS-c levels decrease under T1D conditions (Fig. 2a). Consistent with this previous T1D and MOTS-c study, we found that MOTS-c levels are also lower in serum (Fig. 3b) and β-cells of patients with T2D (Fig. 3a). Aging reduces MOTS-c levels in both the serum and pancreatic β-cells (Fig. 1d,e). Note that MOTS-c levels in 90-week-old pancreatic β-cells are 14-fold lower than those in 12-week-old pancreatic β-cells (Fig. 1d), suggesting that endogenous MOTS-c is largely depleted by aging. A decline in MOTS-c levels associated with aging and diabetes has also been reported in various tissues, including skeletal muscle and circulation, by our group and others^6–10,111^. Based on these three β-cell senescence models, we demonstrated that MOTS-c levels are lower or largely depleted in senescent pancreatic β-cells compared with young or control groups. However, the exact mechanism of MOTS-c or other mitochondrial peptide transport—from mitochondria to the cytosol, from the cytosol to the extracellular matrix or into the circulation—remains largely unknown. Since mitochondria are highly compartmentalized organelles requiring specific transporters for the movement of metabolites and molecules, the transportation of endogenously expressed MOTS-c within organelles by transporters such as SLC25 family and between cells or tissues require further investigation.

The development of senolytics to eliminate senescent cells is one of the strategies for treating age-related diseases^112^. The principle of senolytic approach is to target antiapoptotic pathways by inhibiting or activating proteins that regulate apoptosis resistance^112^. A major challenge in eliminating senescent cells is their intrinsic resistance to apoptotic stimuli making most cytotoxic drugs ineffective^112^. Unlike most senolytics, MOTS-c regulates OXPHOS and stress-related signals such as AMPK or mTORC1 (Figs. 5g, and 6b,c,g), which do not overlap with the targets of other senolytics, and it can be used in combination with other antidiabetic drugs, such as exendin-4 (Fig. 2). There are seven different types of senolytic drug that target BCL-2 proteins (navitoclax and venetoclax), tyrosine kinase/BCL-2 (dasatinib/quercentin), antioxidants (fisetin), HSP90 inhibitors (17-DMAG/IPI504), the p53 pathway (UBX0101/Foxo4-DRI peptide), Na^+^/K^+^ ATPase (cardiotonic steroids) and galactosidase prodrugs (GMD/SSK1)^112^. Unlike most senolytics, which are synthetic drugs, MOTS-c is naturally conserved across many species^8^. Furthermore, MOTS-c treatment can be administered intermittently in higher concentrations^9^, which exhibited similar effects to daily treatments in mice^9^. This suggests that MOTS-c, as a drug, is relatively safe to use alone or in combination. However, further investigations are needed regarding the dosage, selectivity and toxicity of MOTS-c in humans.

In summary, we found that MOTS-c is reduced with aging and senescence in β-cells and the extent of MOTS-c levels upon oxidative stress declines. Using three established rodent models of β-cell senescence, we show that MOTS-c not only reduces cellular signatures of cellular senescence in pancreatic islets but also delays the onset of diabetes. In this study, we showed that systemic MOTS-c treatment in two different pathophysiological mechanisms of diabetes in mouse models (β-cell senescence in NOD mice and insulin-antagonist S961-induced insulin-resistant C57BL/6 mice) can reduce senescence in pancreatic β-cells and delay the onset of diabetes.

## Supplementary information

Supplementary Information

Supplementary Table 1

Supplementary Table 2

Supplementary Table 3

Supplementary Table 4

## Data Availability

Pancreatic islet microarray data have been deposited in GEO under accession code GSE305383.
