# Supplementary material for: Mitochondrial-encoded peptide MOTS-c prevents pancreatic islet cell senescence to delay diabetes
Source: Exp Mol Med. 2025 Aug 25;57(8):1861–77. doi: 10.1038/s12276-025-01521-1 (PMC12411631; doi:10.1038/s12276-025-01521-1)
Supplement: Supplementary file 1 — Supplementary Information [file 12276_2025_1521_MOESM1_ESM.pdf]

a b

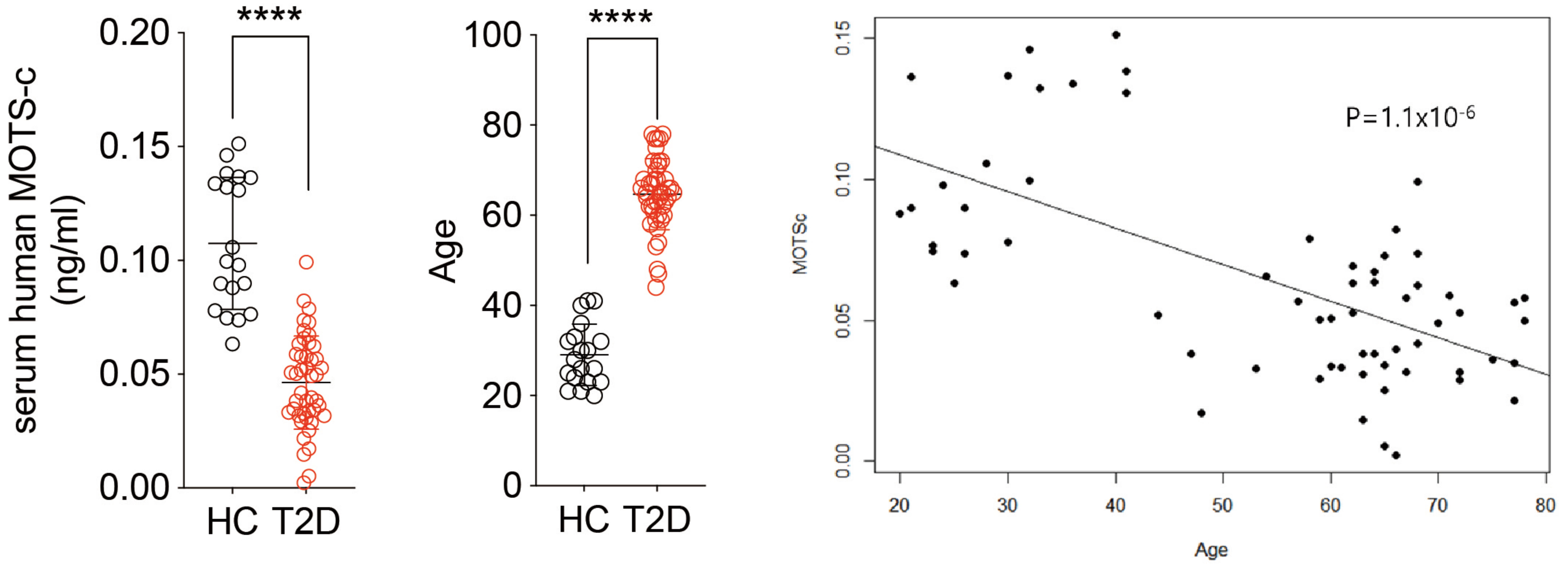

c

	nonT2D	T2D	P	P*
N	19	45		
MOTS-c (SD)	0.11	0.05	$6.7x10^{-14}$	$4.4x10^{-14}$
Age (mean, SD)	29.1 (6.8)	64.7 (7.9)	<0.001	
Sex (Female, %)	5 (26)	23 (51)	0.121	

P ; tested for ANOVA
P* ; adjusted for age and sex using ANCOVA

Supplementary Fig. 1. related to Figure 1.
(**a**) T2D (n=45) and healthy control (n=19) serum were compared for age and circulating MOTS-c level (Figure 3b) at fasting baseline. Two-tailed t-test; error bars are SD. ****p<0.0001.

(**b-c**) To identify the correlation between age and MOTS-c level in healthy controls and T2D patients, (**b**) Spearman rank correlation was performed. The difference in mean of serum MOTS-c level between healthy control and diabetes group adjusted by age was compared using ANCOVA. (**c**) The participant characteristics are shown in table.

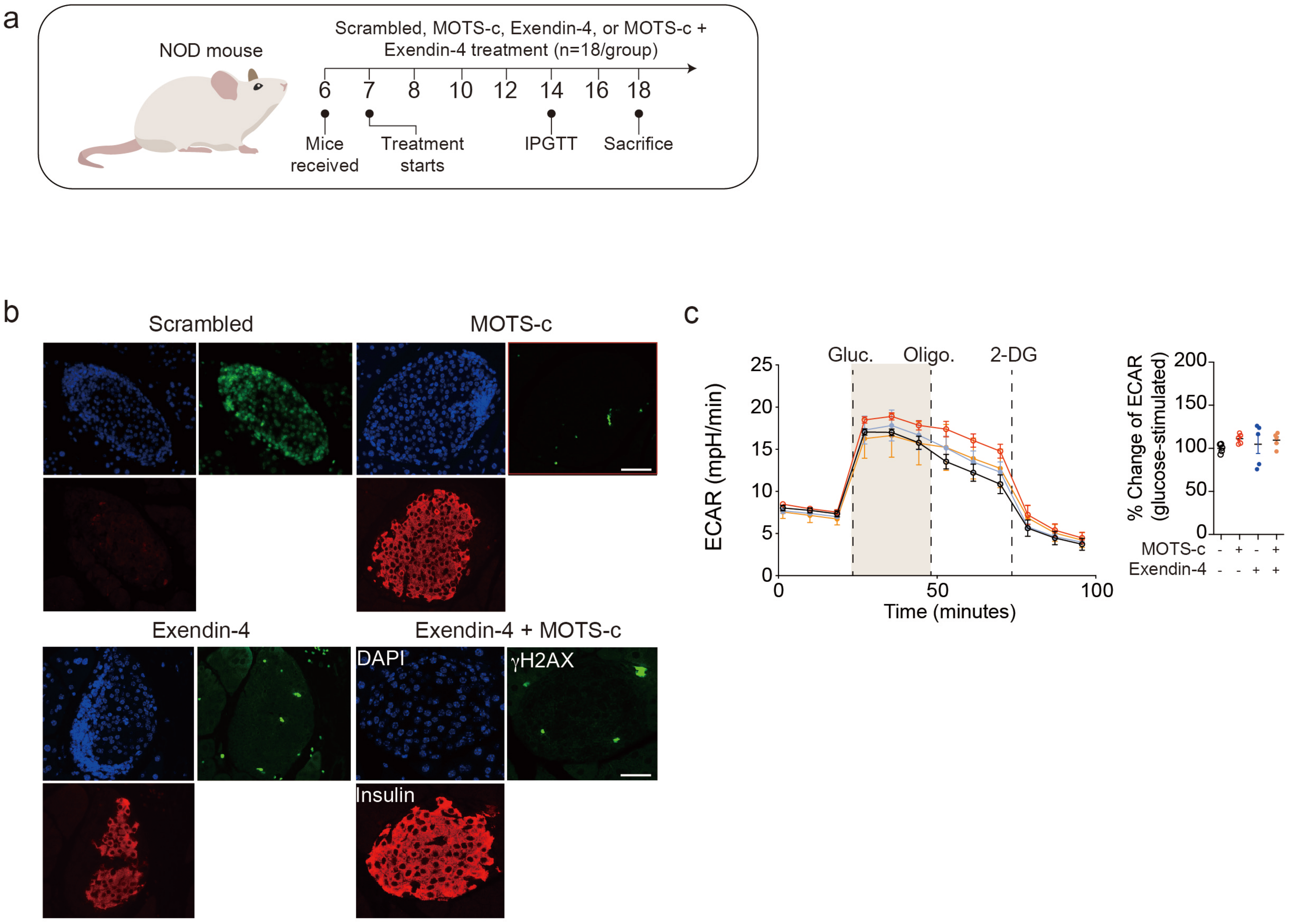

Supplementary Fig. 2. related to Figure 2.
(**a**) A diagram illustrating the timeline of MOTS-c and exendin-4 treatment in NOD mice.
(**b**) Immunofluorescence staining was performed on pancreas tissues from 18-week-old NOD to analyze γ-H2AX and Insulin expression. Scale bars: 50 μm.
(**c**) Pancreatic islet cells isolated from 18-week-old NOD mice (n=5/group) were analyzed for ECAR (extracellular acidificication rate) using metabolic flux analyzer.

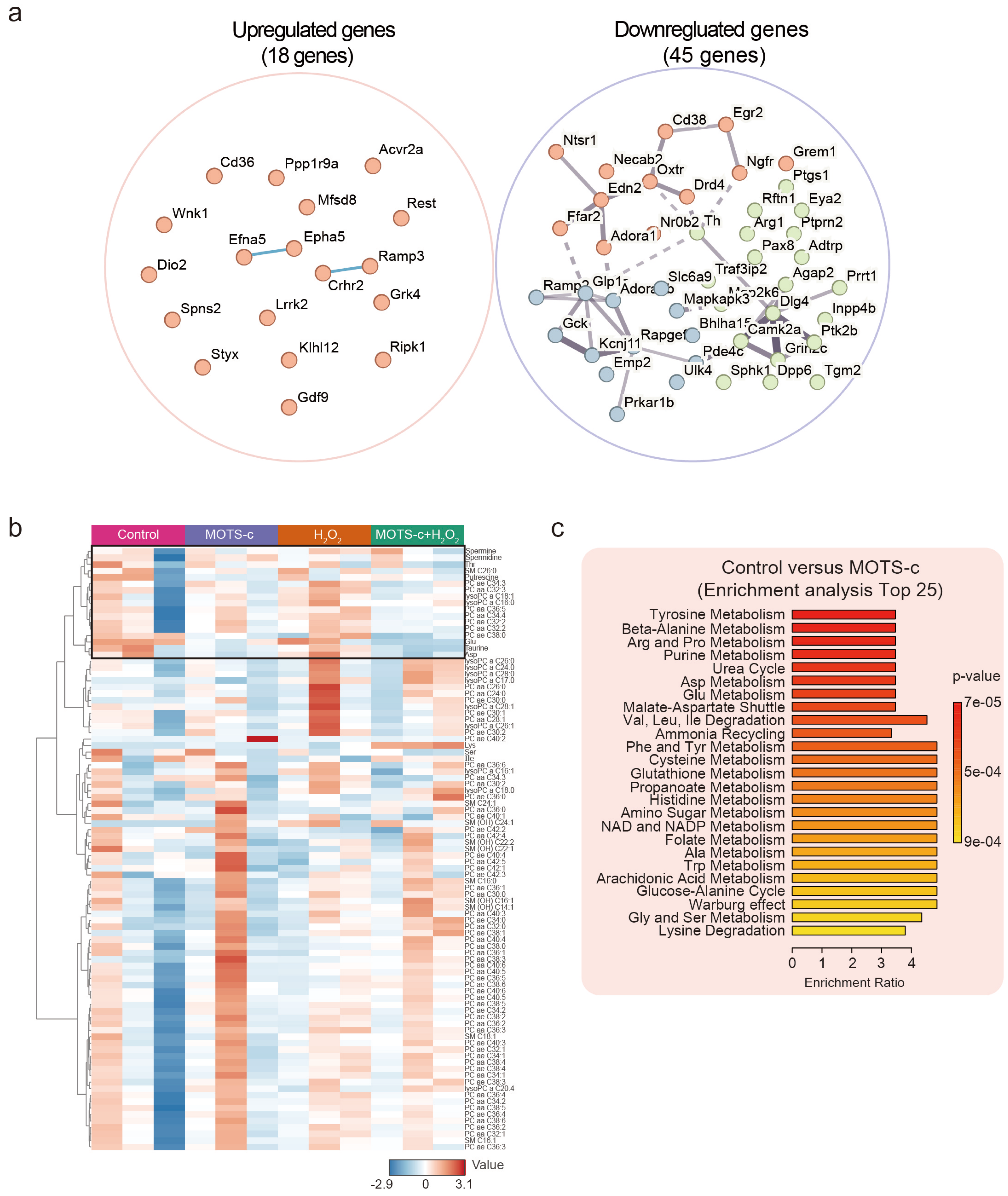

Supplementary Fig. 3. related to Figure 4.

(**a**) Commonly shared genes (**Fig. 4g**) were analyzed and clustered with STRING analysis. Downregulated genes are clustered in 3 different nodes (red, green, blue) using k-means clustering. Blue node genes (insulin secretion-related), red node genes (calcium and cAMP signaling), and green node genes (NMDA glutamate receptor complex) are annotated and clustered based on KEGG pathways (**Fig. 4c**). The thickness of lines between each node indicates the strength of interaction between proteins. Dotted line shows less interactions between proteins.

(**b**) Pancreatic islet cells were isolated from littermates of 60-week-old C57BL/6 mice (n=3/group). Then, pancreatic islet cells were treated with either MOTS-c, scrambled peptide (10 μM, 24h), and hydrogen peroxide (200 μM, 24h). Metabolites were isolated to perform metabolomics and shown in heat-map. Black box indicates zoom-in data for **Fig. 5b**. Enrichment analysis were performed between (**c**) control vs MOTS-c and (**Fig. 4c**) H_2O_2 versus MOTS-c+H_2O_2.

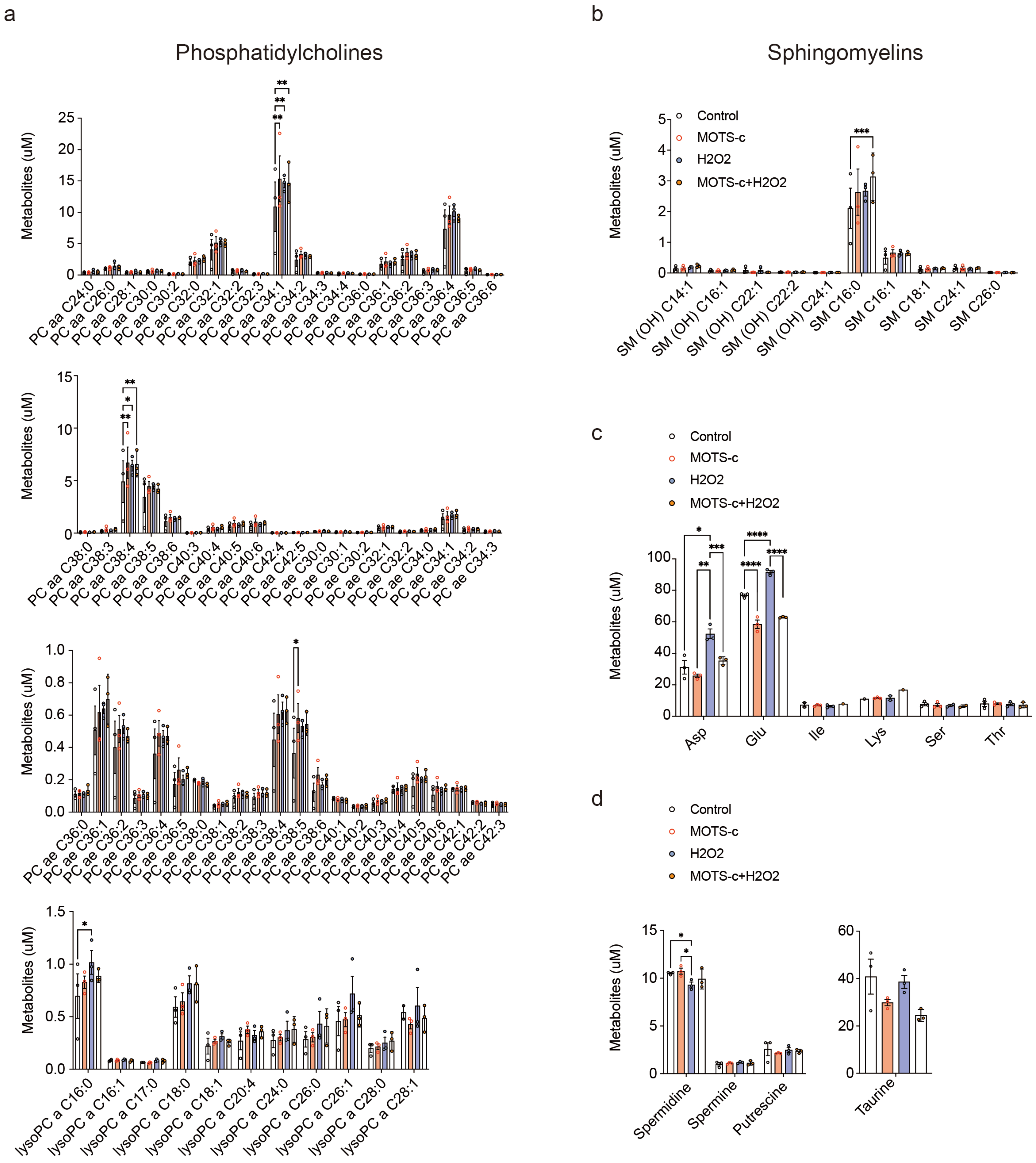

Supplementary Fig. 4. related to Figure 5.
MOTS-c or scrambled control (10 μM, 24h) and hydrogen peroxide (200 μM, 24h) treatment in pancreatic islet cells (n=2 mice/sample) isolated from littermates of 60-week-old C57BL/6 mice (n=3/group). 188 metabolites associated with (**a**) phosphatidylcholines, (**b**) sphingomyelins, (**c**) amino acids, and (**d**) taurine were measured.

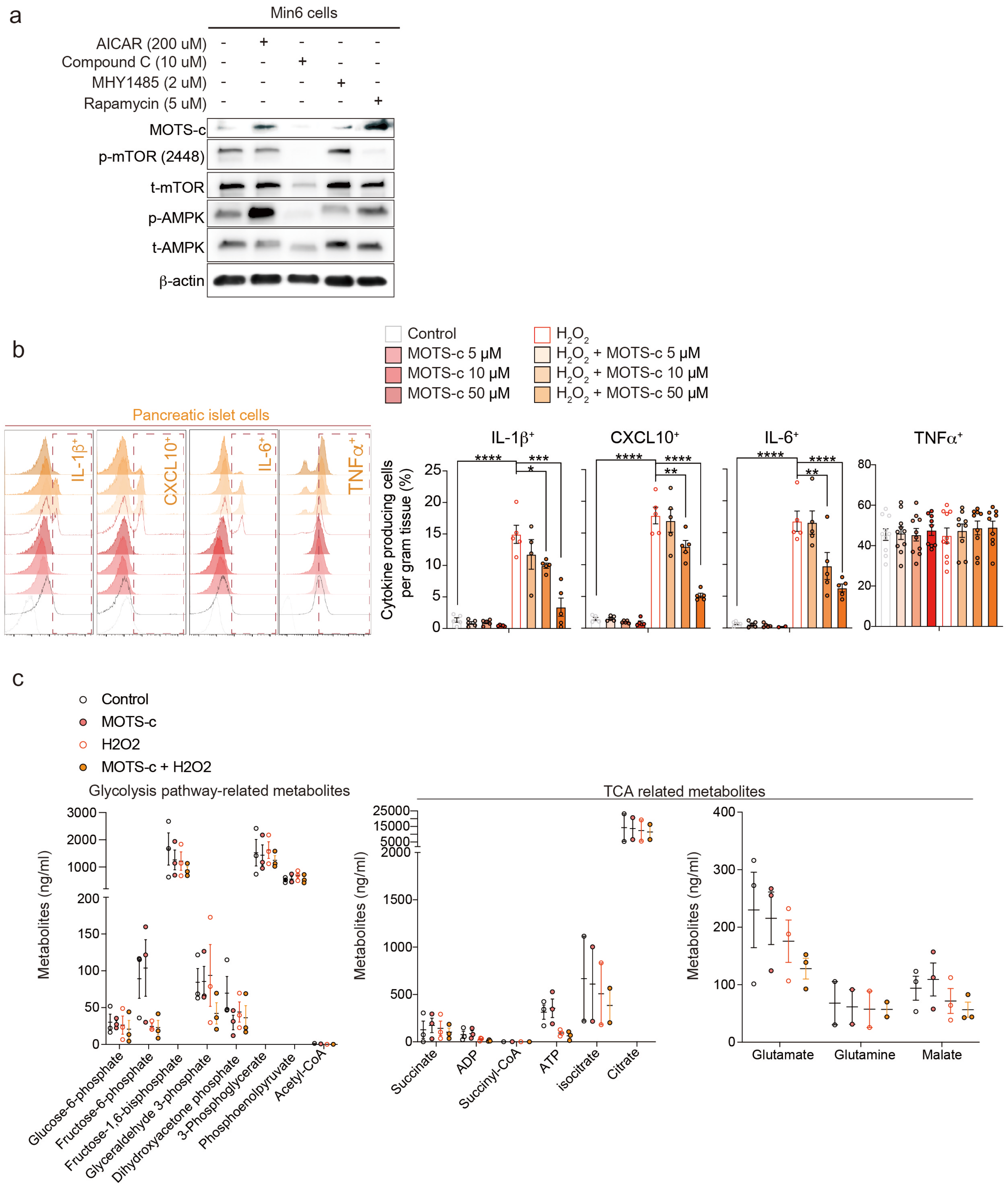

Supplementary Fig. 5. related to Figure 6.

(**a**) To understand the relationship between MOTS-c, AMPK, and mTORC1, Min6 cells were treated with AMPK activator (AICAR, 200 μM, 24h), AMPK inhibitor (compound c, 10 μM, 24h), mTOR activator (MHY1485, 2 μM, 24h), and mTOR inhibitor (Rapamycin, 5 μM, 24h). Then, MOTS-c, p-mTOR(2448), t-mTOR, p-AMPK, t-AMPK, and β-actin were analyzed.

(**b**) Pancreatic islet cells were isolated from 12-week-old C57BL/6 mice to treat with MOTS-c (5, 10, 50 μM, 24h) and/or H_2O_2 (200 μM, 24h). Then, cells were stained and analyzed for IL-1β, Cxcl10, IL-6, and TNFα. Two-way ANOVA, error bars are SEM. *p<0.05, **p<0.01, ***p<0.001, ****p<0.0001.

(**c**) LC-MS/MS analysis (n=3, 5×10^5 cells/sample) was performed to identify the role of MOTS-c during H_2O_2-induced senescence in Min6 cells. Min6 cells were treated with or without MOTS-c (10 μM) and/or H_2O_2 (200 μM) for 24 hours. Then, whole cells were acquired for targeted metabolomics to identify metabolites involved in glycolysis (glucose-6-phosphate, fructose-6-phosphate, fructose-1,6-biphosphate, glyceraldehyde 3-phosphate, dihydroxyacetone phosphate, 3-phosphoglycerate, phosphoenolpyruvate, and acetyl-CoA) and TCA cycle (succinate, ADP, ATP, succinyl-CoA, isocitrate, glutamate, glutamine, and malate).
